# Soft‐Drug‐Inspired MnSTF Nano‐Adjuvant for Safe and Synergistic cGAS–STING Activation in Tumor Immunotherapy

**DOI:** 10.1002/advs.202515432

**Published:** 2025-12-16

**Authors:** Guangfei Sun, Jiancheng Pan, Rui Li, Ruoxi Li, Ziyan Liu, Jinhui Wu, Tingsheng Lin, Yiqiao Hu, Ahu Yuan

**Affiliations:** ^1^ State Key Laboratory of Pharmaceutical Biotechnology Medical School and School of Life Science Nanjing University Nanjing 210093 P. R. China; ^2^ Department of Urology Nanjing Drum Tower Hospital The Affiliated Hospital of Nanjing University Medical School Nanjing 210093 P. R. China; ^3^ Jiangsu Key Laboratory for Nano Technology Nanjing University Nanjing 210093 P. R. China

**Keywords:** antigen‐specific T cell response, nanoadjuvant, soft‐drug design, STING pathway, tumor immune microenvironment

## Abstract

The activation of the stimulator of interferon genes (STING) pathway is a cutting‐edge strategy in tumor immunotherapy and has shown transformative potential in preclinical studies by inducing a type I interferon cascade and remodeling the tumor immune microenvironment. However, current STING agonists are limited by a narrow therapeutic window and substantial systemic toxicity, which significantly impedes their translational potential. To overcome these challenges, a novel self‐assembling nanoadjuvant, MnSTF, grounded in the soft‐drug design paradigm is designed. MnSTF is assembled through multidentate coordination between manganese ions (Mn^2+^), which exhibit moderate STING‐activating activity, and the ENPP1 inhibitor STF‐1623, thereby achieving synergistic activation of the cGAS–STING signaling axis. In contrast to conventional small‐molecule STING agonists, MnSTF elicits no systemic inflammatory response at either therapeutic or supratherapeutic doses, demonstrating outstanding safety and biocompatibility. Furthermore, MnSTF profoundly reprograms the tumor immune microenvironment and, when coadministered with radiotherapy, mRNA vaccines, or protein antigens, induces robust antigen‐specific T cell responses and marked antitumor efficacy. Accordingly, by combining soft‐drug design with a dual‐targeted immunomodulatory mechanism, MnSTF effectively reconciles the efficacy–safety trade‐off of STING agonists in preclinical settings and expands the potential for precise immunoregulation via the STING pathway.

## Graphical abstract

This study introduces MnSTF, a soft‐drug‐inspired nanoadjuvant that overcomes the systemic toxicity of STING agonists. Through Mn^2+^ coordination with an ENPP1 inhibitor, it enables safe cGAS‐STING activation, remodels the tumor immune microenvironment, and synergizes with radiotherapy or vaccines to elicit robust antitumor immunity. The platform establishes a new paradigm for balancing efficacy and safety in cancer immunotherapy.

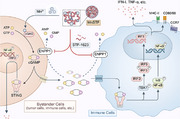

## 1 Introduction

The activation of the STING signaling pathway triggers the synthesis and secretion of type I interferons (IFN‐I) and a broad array of pro‐inflammatory cytokines, promotes dendritic cell (DC) maturation, and enhances both antigen cross‐presentation and antigen‐specific T cell responses.^[^ ^1^, ^2^ ^]^ Furthermore, STING pathway activation remodels the tumour immune microenvironment (TIME), converting immunophenotypically “cold” tumours into “hot” tumours and thereby orchestrating synergistic antitumour responses by the innate and adaptive immune systems.^[^ ^3^, ^4^, ^5^, ^6^ ^]^ As a pivotal target in tumor therapy, the cGAS–STING axis has driven the development of numerous agonists,^[^ ^7^ ^]^ ranging from the endogenous cyclic dinucleotide 2′,3′‐cGAMP^[^ ^8^ ^]^ and its derivative ADU‐S100^[^ ^9^ ^]^ to exogenous small‐molecule agonists such as diABZI,^[^ ^10^ ^]^ SR717,^[^ ^11^ ^]^ and MSA‐2.^[^ ^12^ ^]^ Although these agents exhibit potent immunostimulatory activity in early studies, their clinical translation remains hindered by the challenge of balancing efficacy and safety (e.g., ADU‐S100: NCT03172936; XMT‐2056: NCT05514717). For example, potent agonists such as MSA‐2 lack tissue specificity and may induce systemic STING hyperactivation, leading to dose‐limiting toxicities (DLTs) that significantly restrict their clinical utility.^[^ ^12^ ^]^ Even novel formulations that use conjugation or encapsulation to prolong intravascular release ultimately release the parent active molecule‐a “hard drug”‐into the circulation, where it interacts with pattern‐recognition receptors (PRRs) on diverse immune cells, risking widespread inflammatory responses.^[^ ^13^, ^14^, ^15^ ^]^ In contrast, a “soft drug” is engineered to provide defined bioactivity before undergoing rapid, predictable metabolism into inactive or minimally toxic metabolites, thus minimizing off‐target and cumulative toxicities.^[^ ^16^ ^]^ Inspired by this paradigm, we propose an innovative nanoadjuvant strategy that imparts soft‐drug properties to STING agonists: following local administration and immune activation, the nanoadjuvant undergoes metabolism through distinct pathways into inactive or low‐activity metabolites, thereby preventing sustained systemic immune activation. Concurrently, the nanoscale formulation substantially reduces systemic absorption rates and peak plasma concentrations, further mitigating systemic exposure and immune‐related adverse effects.

To implement the soft‐drug nanoadjuvant design concept outlined above, we modularized the adjuvant into multiple cooperating components to activate immune responses at the target site. When co‐localized, these modules synergistically potentiate immunity (on‐target coupling); once dispersed into systemic circulation, their coupling dissolves, and the individual components are rapidly metabolized and cleared, thereby terminating the synergistic effect (off‐system decoupling). This “on‐target coupling–off‐system decoupling” dynamic control mechanism ensures a favorable safety profile. In 2020, Jiang and colleagues discovered that Mn^2+^ directly binds to cGAS, even in the absence of double‐stranded DNA‐inducing the conformational changes necessary for active dimer formation and the catalysis of the second messenger 2′,3′‐cGAMP, thereby activating STING and its downstream NF‐κB and IRF3 pathways.^[^ ^17^ ^]^ However, they also identified a critical limitation: in the absence of exogenous dsDNA, Mn^2+^ requires millimolar concentrations to elicit substantial cGAS–STING activation. This finding underscores the intrinsic “mild potency” of Mn^2+^ as a natural STING agonist‐despite being mechanistically capable of pathway activation, its standalone immunostimulatory efficiency is low. Conversely, multiple negative regulators inhibit the cGAS–STING axis through distinct mechanisms.^[^ ^18^ ^]^ Notably, Samuel Bakhoum's team at Memorial Sloan Kettering Cancer Center first demonstrated that the extracellular enzyme ENPP1, ubiquitously localized to cell membranes, hydrolyzes 2′,3′‐cGAMP, thereby preventing paracrine activation of neighboring immune cells and promoting tumor immune evasion and metastasis.^[^ ^19^, ^20^, ^21^, ^22^ ^]^ To counteract this checkpoint, STF‐1623 was developed as a cell‐impermeable ENPP1 inhibitor; it exhibits high potency and a short half‐life, enabling rapid clearance upon systemic exposure and providing an excellent safety margin.^[^ ^20^, ^23^ ^]^ Accordingly, in this study, we aim to inhibit ENPP1 and other negative STING modulators to amplify the synergistic activation induced by Mn^2+^, incorporating these elements into a soft‐drug nanoadjuvant that enables controlled release and systemic decoupling. This strategy is designed to maximize local immune activation while minimizing systemic exposure and safety risks.

**Figure 1:**
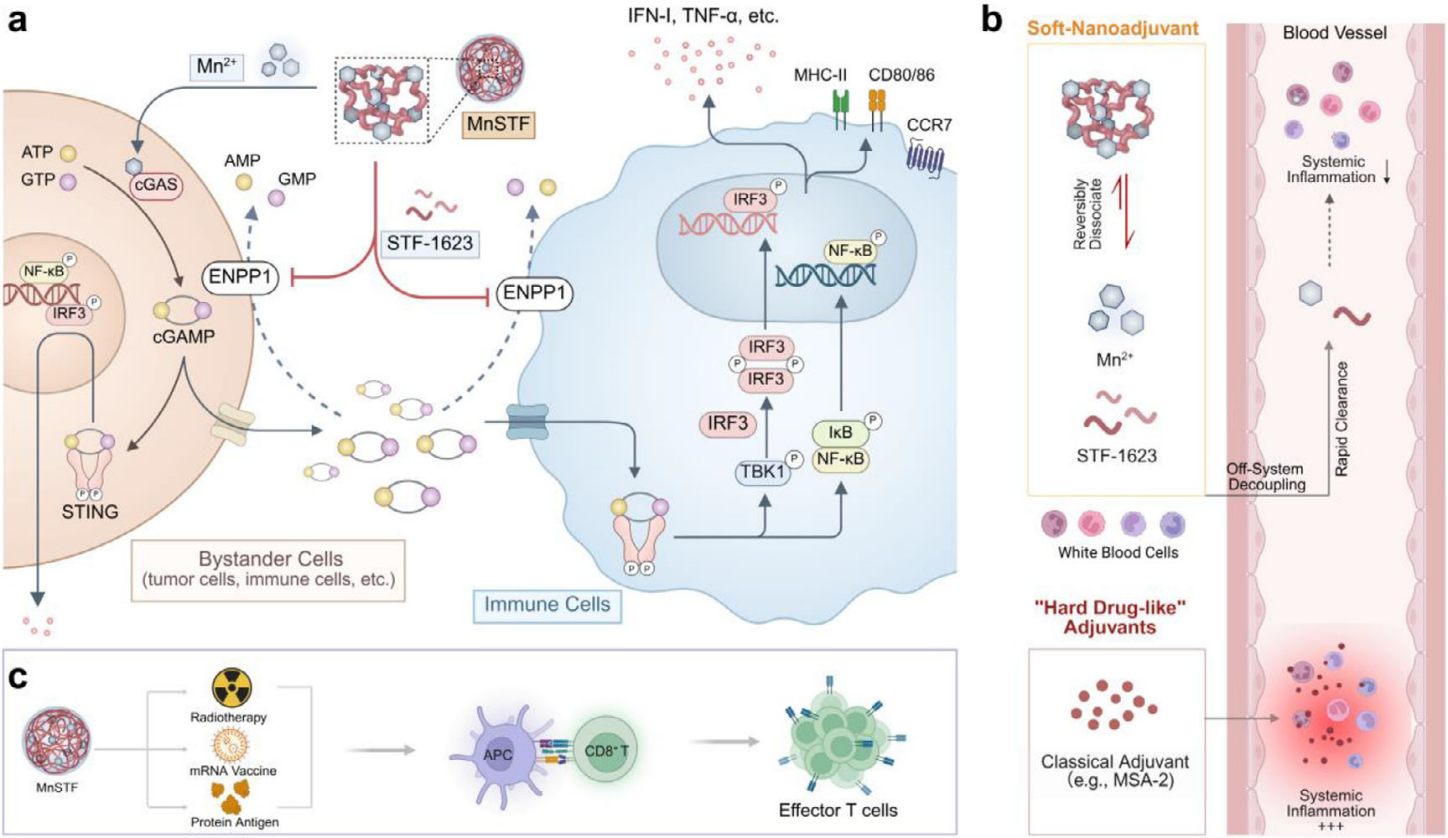
Schematic illustration of a soft drug‐inspired nanoadjuvant platform designed to potentiate antitumor immunity while minimizing off‐target toxicity. (a) Schematic representation of MnSTF potentiating cGAS–STING activation. (b) Mechanistic illustration of MnSTF evading systemic inflammation through coordinated on‐target coupling, off‐system decoupling, and metabolically tuned regulation. (c) Conceptual schematic of MnSTF combined with radiotherapy, mRNA vaccine, or protein antigen synergistically enhancing antitumor immunity by potentiating antigen‐specific T cell responses. Created with BioRender.com.

This study demonstrates that STF‐1623 harbors phosphate groups and electron‐rich nitrogen atoms, enabling strong chelation with Mn^2+^ to form stable multidentate complexes that scaffold an interconnected network and self‐assemble into MnSTF (Mn^2+^–STF‐1623) nanoparticles. The resulting nanoparticles exhibit a negative surface charge, an average diameter of approximately 184 nm with a narrow size distribution, and demonstrate controlled‐release behavior. Mechanistically, MnSTF exerts dual immunomodulatory functions: released Mn^2+^ augments 2′,3′‐cGAMP synthesis, whereas STF‐1623 potently inhibits extracellular ENPP1 activity, preventing 2′,3′‐cGAMP degradation and synergistically increasing extracellular 2′,3′‐cGAMP levels. Downstream signaling analyses revealed that MnSTF significantly enhances the phosphorylation of TBK1, IRF3, and NF‐κB, thereby inducing the expression and secretion of type I interferons (IFN‐I) and multiple pro‐inflammatory cytokines, confirming effective activation of the cGAS–STING pathway. In the B16F10‐OVA tumor model, systemic IL‐6 levels induced by MnSTF at the therapeutic equivalent dose were markedly lower than those induced by the classical small‐molecule STING agonist MSA‐2 (30.6 ± 2.9 pg/mL versus 334.9 ± 51.7 pg/mL), corresponding to a 91% reduction. Even at 5–10× higher doses of MnSTF, no significant elevation of inflammatory cytokines was observed; by contrast, high‐dose MSA‐2 provoked a severe cytokine storm (IL‐6 increased 15.5‐fold), underscoring the safety advantages of MnSTF. Further analysis demonstrated that intratumoral injection of MnSTF significantly enhanced immune cell infiltration and functional reprogramming within the tumor microenvironment (TME): the proportion of CD11c⁺ dendritic cells increased from 2.3% to 7.4% and iNOS^+^F4/80^+^ M1 macrophages increased from 1.2% to 7.6%, representing approximately 3.2‐ and 6.3‐fold increases, respectively. When combined with radiotherapy, MnSTF enhanced dendritic cell cross‐presentation (1.6 ± 0.3% vs 1.0 ± 0.4%) and maturation (CD80^+^CD86^+^: 28.6 ± 3.5% versus 18.2 ± 1.8%), and promoted CD8⁺ T cell activation and tumor infiltration. In combination with mRNA vaccination, MnSTF significantly increased OVA‐specific CD8^+^ T cells (4.1 ± 1.2% versus 1.5 ± 0.4%, ∼2.7‐fold) and induced epitope spreading to multiple tumor antigens (e.g., SVYDFFVWL, SIYRYYGL, KVPRNQDWL). Moreover, compared to an alum adjuvant, MnSTF elicited higher OVA‐specific CD8⁺ T cell responses (203 ± 74 versus 6 ± 3 spots/10^6^ cells). In the tumor‐bearing mouse model, the combination regimen significantly inhibited tumor growth and increased the proportion of effector memory T cells (TEM) compared to OVA monotherapy (44.1 ± 6.7% versus 27.7 ± 4.5%). In summary, by integrating nanostructural self‐assembly with a soft‐drug design, MnSTF enables precise modulation of the STING pathway and substantially enhances the therapeutic index, surmounting the traditional trade‐off between activation potency and systemic toxicity, and offering an extensible design paradigm for next‐generation STING‐agonistic nanoadjuvants (**Figure** **1**).

## 2 Results and Discussion

### 2.1 Soft Drug‐Inspired Nanoadjuvant Platform: Design and Characterization

**Figure 2:**
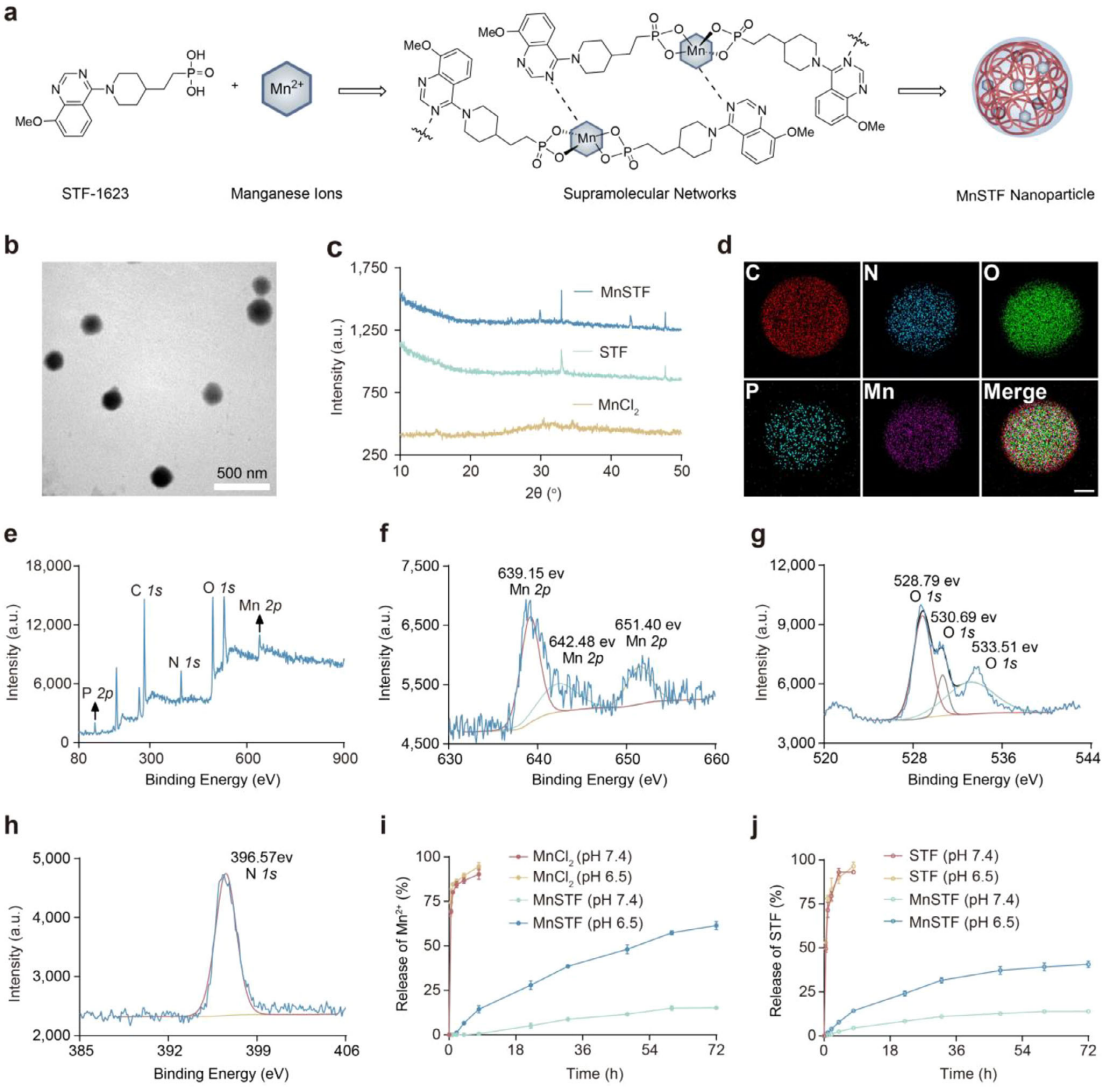
Characterization of the soft drug‐inspired nanoadjuvant platform. (a) Schematic diagram of MnSTF preparation. (b) TEM image of MnSTF. Scale bar, 500 nm. (c) PXRD patterns of MnCl_2_, STF‐1623 (hereafter STF), and MnSTF. (d) EDS elemental mapping of MnSTF. Scale bar, 50 nm. (e) XPS survey spectrum of MnSTF. (f–h) High‐resolution XPS spectra of Mn *2p* (f), O *1s* (g), and N *1s* (h) for MnSTF. (i, j) Cumulative release profiles of Mn^2+^ from MnCl_2_ and MnSTF (i), and STF from free STF and MnSTF (j), incubated in Tris‐HCl buffer (pH 7.4 and 6.5) at 37 °C. Data are shown as mean ± s.d (n = 3).

Inspired by the soft drug concept, a novel nanoadjuvant, MnSTF (Mn^2+^–STF‐1623), was designed and synthesized through a coordination‐driven self‐assembly approach (**Figure** **2**a).^[^ ^24^ ^]^ In aqueous solution, Mn^2+^ ions and STF‐1623 ligands spontaneously self‐assembled into nanoscale MnSTF complexes, which exhibited distinct Tyndall scattering and a zeta potential of –25.50 ± 1.14 mV (Figure S1a,b, Supporting Information). Comprehensive characterization of the MnSTF nanoadjuvant was then performed. Transmission electron microscopy (TEM) and dynamic light scattering (DLS) revealed that MnSTF nanoparticles had a near‐spherical morphology with an average diameter of 184 ± 35 nm (Figure 2b; Figure S1c, Supporting Information). Powder X‐ray diffraction (PXRD) analysis indicated that MnSTF exhibited a polycrystalline structure, implying potential polymorphism (Figure 2c). Energy‐dispersive X‐ray spectroscopy (EDS) mapping confirmed a homogeneous spatial distribution of C, N, O, P, and Mn within the nanocomplex. Complementary X‐ray photoelectron spectroscopy (XPS) analysis of MnSTF also revealed characteristic peaks corresponding to P, C, N, O, and Mn (Figure 2e; Figure S1d,e, Supporting Information). High‐resolution Mn *2p* XPS spectra of MnSTF (Figure 2f) displayed distinct Mn^2+^ binding energy peaks at 639.15 and 651.40 eV, as well as an additional peak at 642.48 eV, indicative of lower reduction‐state Mn species resulting from coordination between Mn^2+^ and STF‐1623.^[^ ^25^ ^]^ Moreover, relative to free STF‐1623 (Figure S1f–j, Supporting Information), MnSTF displayed notable binding energy shifts for both O and N atoms. Specifically, the O *1s* binding energy shifted from 530.28 eV in free STF‐1623 to 528.79, 530.69, and 533.51 eV (Figure 2g), wherase the N *1s* binding energy shifted from 398.00 to 396.57 eV (Figure 2h). These coordination‐induced shifts provided compelling evidence for Mn–O and Mn–N bond formation.^[^ ^26^, ^27^ ^]^

Furthermore, Fourier transform infrared (FTIR) and UV–vis spectra confirmed that MnSTF retained the characteristic absorption peaks of both Mn^2+^ and STF‐1623 (Figure S1k, l, Supporting Information), further supporting their coordination. Under acidic conditions (e.g., 0.2% formic acid), MnSTF rapidly and completely dissociated (Figure S1a, Supporting Information). The released Mn^2+^ and STF‐1623 were analyzed quantitatively by formaldoxime colorimetry^[^ ^28^, ^29^ ^]^ (Figure S1m, Supporting Information) and high‐performance liquid chromatography (HPLC) (Figure S1n, Supporting Information), respectively, confirming a Mn^2+^:STF‐1623 molar ratio of 1:2 within the nanocomplex. The release kinetics of MnSTF were then evaluated and compared with those of the free components. The results demonstrated that free Mn^2+^ and STF‐1623 exhibited a burst release profile, with over 80% released within 2 h. In contrast, MnSTF displayed controlled and sustained release behavior at both pH 7.4 and 6.5, allowing gradual dissociation and release of Mn^2+^ and STF‐1623 (Figure 2i,j). Furthermore, the stability and cellular biocompatibility of MnSTF were evaluated. The results demonstrated that MnSTF remained relatively stable in both 10% serum and PBS (Figure S1o, Supporting Information), and at a Mn concentration of 600 µM, exhibited no significant toxicity toward either RAW264.7 or DC2.4 cells (Figure S1p, Supporting Information).

### 2.2 MnSTF Enhances cGAS‐STING Activation Via Dual Regulation: Inhibition Relief and Positive Feedback Amplification

**Figure 3:**
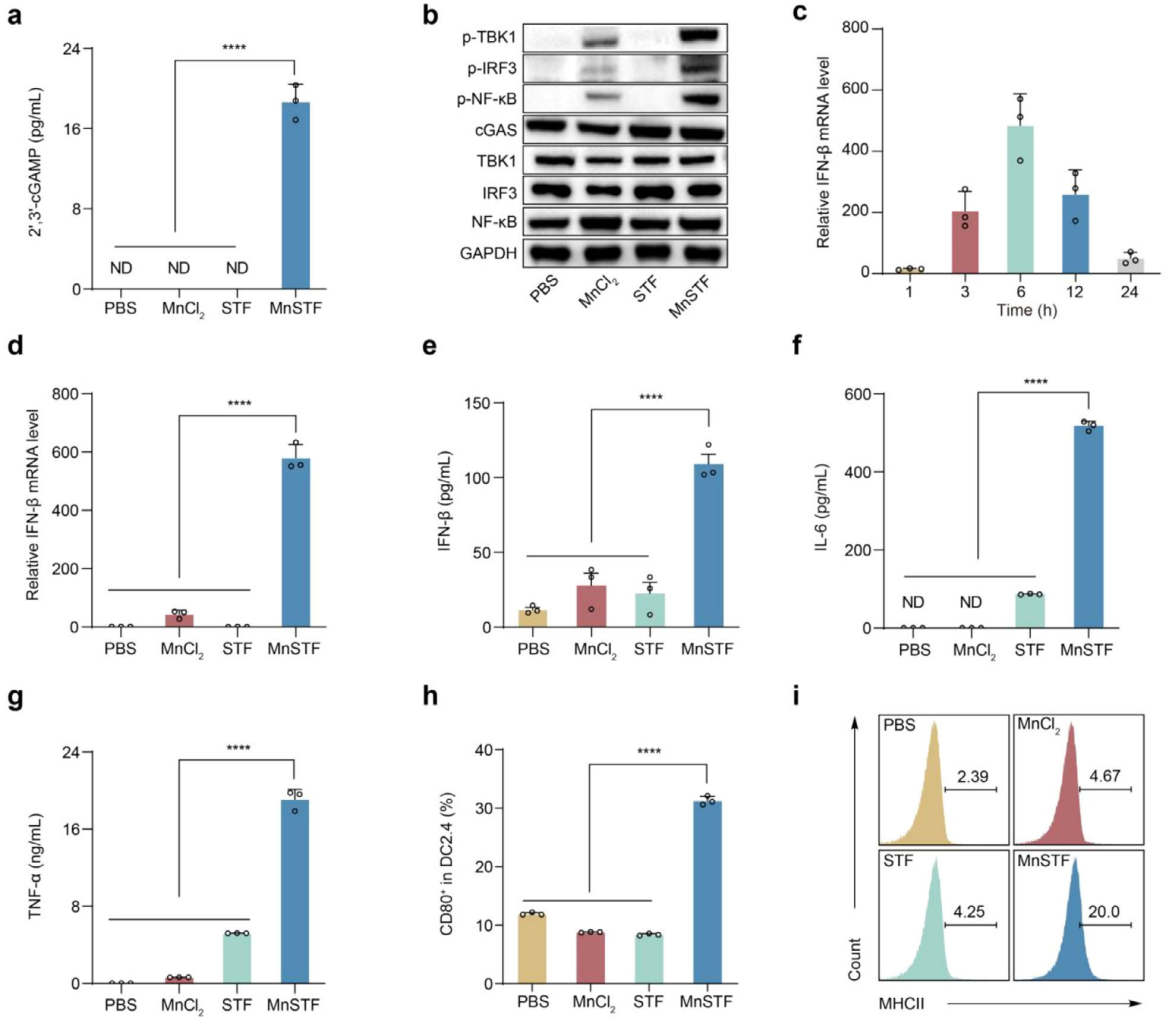
MnSTF augments cGAS‐STING activation. (a) Quantification of extracellular 2′,3′‐cGAMP in RAW264.7 cell supernatants after 24 h treatment, as measured by enzyme‐linked immunosorbent assay (ELISA) (n = 3). ND, not detected. (b) Immunoblot analysis of the cGAS–STING pathway proteins in RAW264.7 cells treated after 6 h treatment. (c) Quantitative real‐time PCR (qRT–PCR) analysis of IFN‐β mRNA levels in RAW264.7 cells treated with MnSTF for the indicated times (n = 3). (d) Relative expression of IFN‐β mRNA in RAW264.7 cells after 6 h treatment (n = 3). (e–g) ELISA quantification of IFN‐β (e), IL‐6 (f), and TNF‐α (g) in culture supernatants of RAW264.7 cells following 24 h treatment (n = 3). (h) Flow cytometric analysis of CD80 surface expression on DC2.4 dendritic cells after 24 h treatment (n = 3). (i) Representative flow cytometric histograms of MHC class II expression on DC2.4 cells treated for 24 h. Replicates were biological and the data were expressed as mean ± s.d. Statistical analysis was determined using one‐way ANOVA, ****p < 0.0001.

Manganese ions are known to activate the cGAS–STING–type I interferon (IFN‐I) pathway;^[^ ^17^, ^30^, ^31^ ^]^ however, 2′,3′‐cGAMP produced by Mn^2+^ alone is rapidly hydrolyzed by ENPP1, which limits its immunostimulatory efficacy.^[^ ^32^ ^]^ MnSTF significantly elevated extracellular 2′,3′‐cGAMP levels (18.7 ± 1.7 pg/mL), whereas free Mn^2+^ or STF‐1623 treatments did not produce detectable 2′,3′‐cGAMP (**Figure** **3**a). Immunoblot analysis further revealed that MnSTF markedly increased the phosphorylation of key downstream signaling molecules, including TBK1, IRF3, and NF‐κB (Figure 3b). Importantly, the expression of p‐IRF3 correlated with IFN‐I production, whereas p‐NF‐κB was associated with induction of pro‐inflammatory cytokines such as IL‐6 and TNF‐α.^[^ ^33^ ^]^ In RAW264.7 cells, MnSTF induced a robust upregulation of IFN‐β mRNA, which peaked at approximately 6 h (Figure 3c), and reached levels over tenfold higher than those induced by free Mn^2+^ (580 ± 46 versus 44 ± 14). In contrast, STF‐1623 alone did not differ significantly from the PBS control (Figure 3d).

ELISA quantification of IFN‐β in culture supernatants confirmed that MnSTF markedly increased IFN‐β production and secretion (109.2 ± 11.2 pg/mL), compared to the minimal levels induced by free Mn^2+^ or STF‐1623 (Figure 3e). A similar trend was observed for pro‐inflammatory cytokines, including IL‐6 and TNF‐α (Figure 3f,g). Moreover, flow cytometry analysis demonstrated that MnSTF markedly upregulated surface expression of the co‐stimulatory molecule CD80 (Figure 3h; Figure S2, Supporting Information) and the antigen‐presenting molecule MHCII (Figure 3i) on DC2.4 cells. Collectively, these in vitro findings demonstrate that MnSTF substantially amplifies Mn^2+^‐mediated cGAS‐STING activation, promotes type I interferons and pro‐inflammatory cytokines production, and effectively drives dendritic cell maturation–highlighting its potential to enhance antitumor immune responses.

### 2.3 MnSTF Exhibits Enhanced In Vivo Biosafety and Biocompatibility

**Figure 4:**
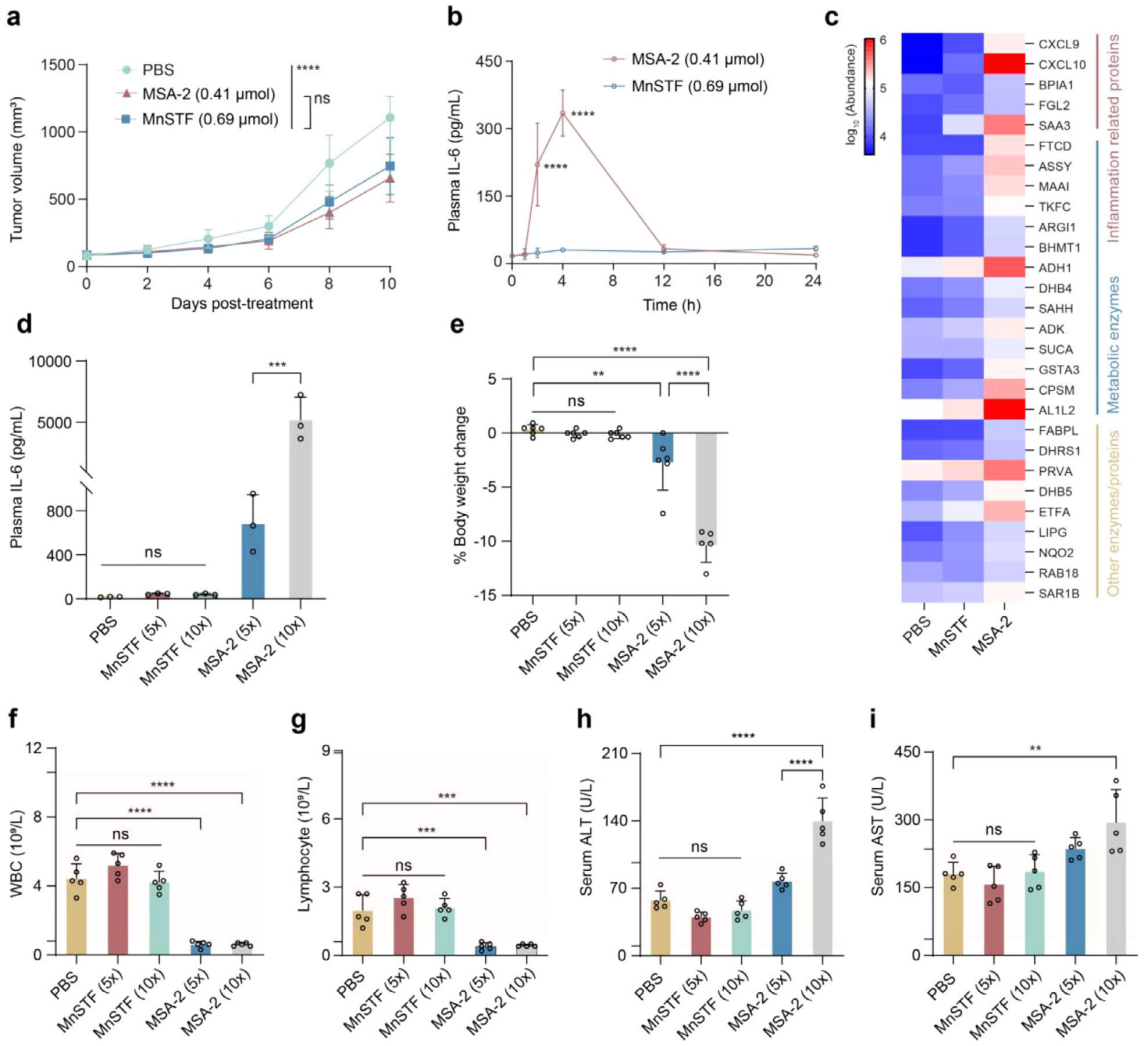
MnSTF shows favorable biocompatibility in vivo. (a) Comparison of antitumor efficacy in C57BL/6 mice bearing B16F10‐OVA tumors after a single intratumoral injection of PBS, MSA‐2 (0.41 µmol), or MnSTF (0.69 µmol Mn equivalent) (n = 6). (b) Time‐course of plasma IL‐6 in C57BL/6 mice after subcutaneous administration of MSA‐2 (0.41 µmol) or MnSTF (0.69 µmol Mn equivalent), quantified by ELISA at indicated time points (n = 3). (c) Proteomic heatmap of plasma cytokines and liver‐associated metabolic enzymes in C57BL/6 mice 4 h after subcutaneous administration of MSA‐2 or MnSTF. (d–i) C57BL/6 mice were administered subcutaneous pulse injections of MSA‐2 or MnSTF at 5× and 10× the therapeutic dose, respectively. (d) Plasma IL‐6 levels in mice at 4 h post‐treatment, quantified by ELISA (n = 3). (e) Percentage of body weight change of mice at 24 h post‐treatment (n = 5–6). (f, g) Peripheral blood parameters at 24 h post‐treatment, white blood cells (WBC) (f) and lymphocytes (g) (n = 5). (h, i) Serum levels of hepatic transaminases: alanine aminotransferase (ALT) (h) and aspartate aminotransferase (AST) (i), (n = 5). Pink bands denote SPF mouse reference ranges (WBC: 0.8–10.6 × 10^9^/L; lymphocytes: 0.6–8.9 × 10^9^/L; ALT: 10.06–96.47 U/L; AST: 36.31‐235.48 U/L). Data were expressed as mean ± s.d. Statistical analysis was performed using two‐way ANOVA (a, b) or one‐way ANOVA (d–i). Statistical significance: **p < 0.01, ***p < 0.001, ****p < 0.0001, ns, not significant.

Immunotherapeutic agents, particularly pattern recognition receptor (PRR) agonists and cytokine‐based therapies, are often limited by narrow therapeutic windows in both preclinical and clinical settings, primarily because of excessive or uncontrolled immune activation.^[^ ^34^, ^35^ ^]^ A comprehensive in vivo toxicological evaluation was conducted to assess the safety profile of MnSTF, a nanoadjuvant rationally designed according to a soft drug strategy. The well‐characterized small‐molecule STING agonist MSA‐2 served as a benchmark for direct comparison. Initially, equivalent antitumor doses of MnSTF (0.69 µmol Mn/mouse) and MSA‐2 (0.41 µmol/mouse) were determined by intratumoral administration in B16F10‐OVA tumor‐bearing mice (**Figure** **4**a). Following administration at these equipotent doses, plasma IL‐6 levels, a hallmark biomarker of cytokine storm,^[^ ^36^ ^]^ were continuously monitored over a 24‐h period after subcutaneous injection. MSA‐2 induced a rapid and substantial increase in systemic IL‐6, reaching a peak concentration of 334.9 ± 51.7 pg/mL within 4 h. In contrast, MnSTF treatment resulted in consistently low and stable IL‐6 levels throughout the observation period, remaining near 30 pg/mL without significant fluctuations (Figure 4b). To comprehensively evaluate the systemic immune responses and potential organ toxicity associated with distinct types of STING agonists, proteomic analysis of plasma samples was performed to quantify a broad panel of cytokines and liver‐associated metabolic enzymes (Figure 4c). The findings revealed that, in contrast to MnSTF, MSA‐2 provoked a pronounced elevation of multiple cytokines in plasma, especially robust upregulation of CXCL9 and CXCL10, suggesting that MSA‐2 has a higher propensity to induce cytokine release syndrome (CRS). Additionally, MSA‐2 treatment significantly elevated the levels of proteins closely associated with acute inflammatory responses, such as serum amyloid A3 (SAA3).^[^ ^37^ ^]^ Notably, proteomic profiling of liver‐associated metabolic enzymes showed marked upregulation in the MSA‐2 group of several enzymes implicated in liver injury, including arginase 1 (ARG1)^,[^ ^38^ ^]^ alcohol dehydrogenase 1 (ADH1)^,[^ ^39^ ^]^ and S‐adenosylhomocysteine hydrolase (SAHH).^[^ ^40^ ^]^ These aberrant alterations are closely associated with the pathological mechanisms underlying drug‐induced liver toxicity.

To ensure an optimal therapeutic index, pulse‐dose safety^[^ ^41^ ^]^ evaluations were conducted at 5‐ and 10‐fold the therapeutic dose via subcutaneous administration, with plasma IL‐6 levels measured 4 h post‐dose. MSA‐2 induced a dose‐dependent sharp increase in IL‐6 levels (5× dose: 683.6 ± 264.1 pg/mL; 10× dose: 5196.4 ± 1841 pg/mL), whereas IL‐6 levels in all MnSTF dose groups remained within the physiological range (<50 pg/mL) (Figure 4d). Over the 24‐h post‐administration period, body weight remained stable in MnSTF‐treated mice, whereas MSA‐2 treatment caused significant dose‐dependent weight loss (5× dose: ‐2.8 ± 2.5%; 10× dose: ‐10.4 ± 1.6%) (Figure 4e), with one death likely attributable to cytokine release syndrome. Hematological (Figure 4f,g) and biochemical (Figure 4h,i) analyses further substantiated these results. High‐dose MSA‐2 administration led to a marked reduction in circulating leukocytes and lymphocytes beyond physiological ranges, consistent with previous reports of 2′,3′‐cGAMP‐induced CD45^+^ cell depletion.^[^ ^42^ ^]^ Concurrently, serum levels of AST and ALT were significantly elevated, indicative of hepatocellular injury. In contrast, MnSTF‐treated mice exhibited no detectable changes in blood cell counts or hepatic enzyme levels. Taken together, these results confirm the exceptional safety and therapeutic index of MnSTF, which does not induce cytokine storm or systemic toxicity even at supra‐therapeutic doses, consistent with its rational soft drug–based design. The long‐term safety evaluation of MnSTF showed no substantial manganese accumulation in major organs 14 days after a three‐dose regimen, as manganese concentrations returned to baseline levels in most organs (Figure S3, Supporting Information). This efficient systemic clearance suggests a reduced risk of Mn‐induced neurotoxicity.

### 2.4 MnSTF Releases In Vivo and Reprograms the Tumor Immune Microenvironment

The immunosuppressive tumor microenvironment (TME), marked by poor effector cell infiltration and diminished proinflammatory cytokine secretion, constitutes a major barrier to effective antitumor immunity, thereby promoting tumor progression, metastasis, and immune evasion.^[^ ^43^ ^]^ To address this challenge, we systematically explored the therapeutic potential of MnSTF‐mediated synergistic cGAS–STING activation for in vivo TME reprogramming.

**Figure 5:**
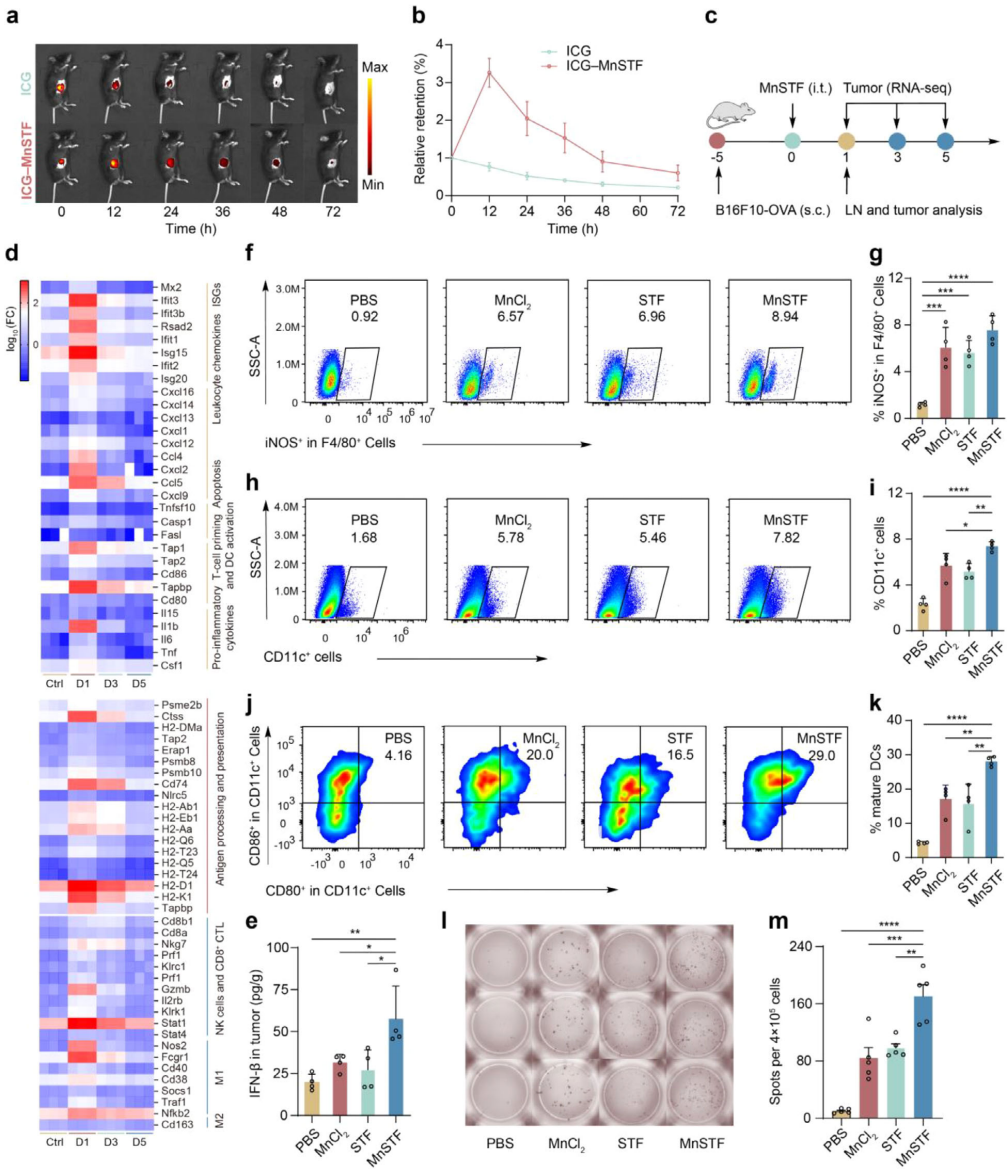
MnSTF regulates the tumor microenvironment by enhancing intratumoral immune responses and reshaping immune cell composition. (a) Representative IVIS fluorescence images of B16F10‐OVA tumor‐bearing mice illustrating intratumoral retention of free ICG or ICG–MnSTF following intratumoral injection. (b) Quantification of intratumoral ICG retention over time (n = 4). (c) Treatment timeline for B16F10‐OVA tumor‐bearing mice administered a single intratumoral injection of PBS or MnSTF (0.69 µmol Mn equivalent), initiated upon tumor palpation (typically 50–100 mm^3^). (d) Heat map of selected genes in tumors on days 1, 3, and 5 post‐treatment, generated from transcriptome sequencing (n = 3). (e) Tumor IFN‐β levels on day 1 post‐treatment, measured by ELISA (n = 4). (f–i) Representative flow cytometry plots and quantification of F4/80^+^iNOS^+^ (f, g) and CD11c^+^ (h, i) cells within tumor tissues at day 1 post‐treatment (n = 4). (j, k) Representative flow cytometry plots (j) and quantification (k) of CD80^+^CD86^+^ expression on CD11c^+^ cells in the draining lymph nodes (LNs) on day 1 post‐treatment (n = 4). (l, m) Representative ELISpot images of IFN‐γ–secreting splenocytes (l) and quantification of spot‐forming units (SFU) per 4 × 10^5^ splenocytes (m) on day 7 after three PBS, MnCl_2_, STF, or MnSTF doses (every three days, n = 5). Replicates were biological, and the data were expressed as mean ± s.d. Statistical analysis was performed using one‐way ANOVA (e, g, i, k, m). Statistical significance: *p < 0.05, **p < 0.01, ***p < 0.001, ****p < 0.0001.

Initially, to characterize in situ release kinetics, ICG‐labeled MnSTF (ICG–MnSTF) was synthesized by incorporating indocyanine green (ICG) during nanoparticle self‐assembly.^[^ ^44^ ^]^ In B16F10‐OVA tumor‐bearing C57BL/6 mice, IVIS imaging demonstrated that ICG–MnSTF displayed a distinct fluorescence profile compared to free ICG. Whereas free ICG rapidly decayed, ICG–MnSTF exhibited a pronounced fluorescence increase within 12 h post‐intratumoral injection, likely due to the initial quenching of ICG within the nanoparticle core followed by fluorescence recovery upon disassembly.^[^ ^45^ ^]^ Sustained fluorescence reduction from 12 to 72 h indicated gradual and prolonged release of MnSTF within tumor tissues (**Figure** **5**a,b). Subsequently, to elucidate the underlying immunomodulatory mechanisms (Figure 5c), we performed time‐resolved transcriptomic profiling after a single intratumoral dose of PBS or MnSTF. Tumors were harvested on days 1, 3, and 5 for RNA‐seq analysis after treatments. Volcano plots revealed a strong early transcriptional response, with 1658 genes upregulated on day 1, followed by progressive attenuation in gene activation (Figure S4a–c, Supporting Information). Gene enrichment (Figure S4d–f, Supporting Information) and Gene Set Enrichment Analysis (GSEA) (Figure S5a, Supporting Information) analysis demonstrated pronounced activation of immune‐ and inflammation‐related pathways, including TNF signaling, chemokine signaling, cytokine–cytokine receptor interactions, and apoptosis. To further dissect the immune remodeling, key differentially expressed genes (DEGs) were grouped into different functional modules (Figure 5d).^[^ ^46^, ^47^ ^]^ The first module represented substantial TME reshaping, characterized by elevated expression of interferon‐stimulated genes (ISGs), proinflammatory cytokines, chemokines, and genes associated with dendritic cell (DC) maturation, T cell activation, and apoptosis. The remaining modules reflected enhanced antigen processing and presentation, with upregulation of genes related to CD8^+^ cytotoxic T lymphocyte function and macrophage polarization toward an M1‐like phenotype. These transcriptomic findings, together with immune cell deconvolution analysis (Figure S5b, Supporting Information), collectively demonstrated a profound reprogramming of the tumor immune microenvironment.

In the tumor microenvironment, type I interferons (IFN‐I), dendritic cells (DCs), and M1‐like macrophages serve as key immunoregulatory mediators and effectors in initiating antitumor immunity.^[^ ^48^, ^49^ ^]^ However, the activity of these proinflammatory mediators and immune cells is often restricted by tumor‐associated immunosuppressive mechanisms.^[^ ^50^ ^]^ Accordingly, we focused on evaluating MnSTF's regulatory effects on intratumoral IFN‐I responses, macrophages, and dendritic cells. Specifically, compared to the PBS control group, MnSTF‐mediated activation of the STING pathway markedly elevated intratumoral IFN‐β levels (Figure 5e). In the immunosuppressive tumor microenvironment, M1‐like macrophages (F4/80⁺iNOS⁺) comprised only 1.2 ± 0.2% in PBS group, but significantly increased to 7.6 ± 1.2% after MnSTF treatment (Figure 5f,g and Figure S6a, Supporting Information), likely attributed to NF‐κB activation and the release of proinflammatory cytokines such as TNF‐α.^[^ ^51^ ^]^ Tumor‐infiltrating CD11c⁺ DCs also increased significantly (7.4 ± 0.4% versus 2.3 ± 0.5%) (Figure 5h,i and Figure S6b, Supporting Information), potentially driven by chemokines such as CCL4 and CCL5.^[^ ^47^ ^]^ Furthermore, we observed that MnSTF induced the repolarization of intratumoral macrophages from the M2 (F4/80⁺CD206⁺) to the M1 (F4/80⁺CD86⁺) phenotype (Figure S6c,d, Supporting Information), which confirmed its role in reversing immunosuppression. It is noteworthy, however, that the same treatment did not yield a significant reduction in Tregs (Figure S6e,f, Supporting Information). Moreover, MnSTF promoted DC maturation via multiple pathways, resulting in a significant increase in the proportion of mature DCs within the tumor‐draining lymph nodes (28.1 ± 1.4%) (Figure 5j,k and Figure S7a, Supporting Information). Finally, to assess systemic antigen‐specific T cell responses induced by MnSTF‐mediated TME remodeling,^[^ ^52^ ^]^ B16F10‐OVA‐bearing mice were immunized with MnSTF three times. Splenocytes harvested 7 days after the final dose were stimulated with OVA_(257‐264)_ peptide, which revealed a robust antigen‐specific T cell response (Figure 5l,m). These findings collectively suggest that MnSTF not only reshapes the local immune landscape by promoting APC recruitment and proinflammatory cytokine production but also serves as a critical link between innate sensing and adaptive T cell priming.

### 2.5 MnSTF Combined with Radiotherapy Enhances Immune Priming and Elicits Systemic Antigen‐Specific T Cell Responses

**Figure 6:**
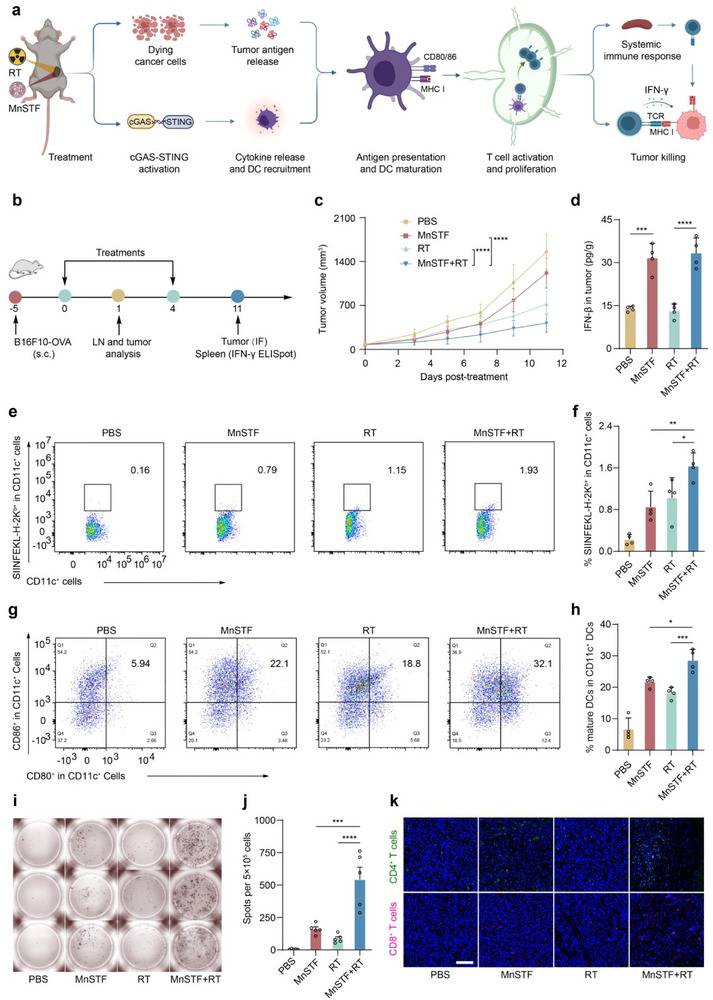
MnSTF synergizes with radiotherapy to enhance immune priming and elicit a robust systemic antigen‐specific T cell response. (a) Schematic illustration of enhanced antitumor immunity induced by RT combined with MnSTF. (b) Experimental timeline for B16F10‐OVA tumor‐bearing mice treated with PBS, RT (6 Gy), MnSTF (0.69 µmol Mn equivalent), or their combination (RT + MnSTF). Treatments were administered every four days for two doses. (c) Tumor growth kinetics following different treatments (n = 9). (d) Tumor IFN‐β levels on day 1 post‐treatments, measured by ELISA (n = 4). (e–h) Representative flow cytometry plots and quantification of CD11c^+^ DCs presenting SIINFEKL–H‐2K^b^ (e, f) and expressing CD80 and CD86 (g, h) in TDLNs on day 1 post‐treatment (n = 4). (i, j) Representative IFN‐γ ELISpot images (i) and quantification of SFU per 5 × 10^5^ splenocytes (j) from each group at the end of treatments (n = 5). (k) Representative immunofluorescence images of tumors at treatment end, stained for CD4^+^ (green) and CD8^+^ (red) T cells, and DAPI (blue). Scale bar, 120 µm. (n = 3). Replicates were biological and the data were expressed as mean ± s.d. Statistical analysis was performed using two‐way ANOVA (c) or one‐way ANOVA (d, f, h, j). Statistical significance: *p < 0.05, **p < 0.01, ***p < 0.001, ****p < 0.0001.

Although radiotherapy (RT) remains a cornerstone in cancer treatment and can moderately suppress tumor growth, its capacity to elicit robust systemic immune responses is limited, primarily because of insufficient dendritic cell infiltration and suboptimal type I interferon (IFN‐I) production within the tumor microenvironment.^[^ ^53^, ^54^, ^55^ ^]^ Given the immunomodulatory properties of MnSTF, including its ability to enhance DC recruitment, drive induction of iNOS⁺ M1‐like macrophage polarization, and increased IFN‐I production, the combinatorial therapeutic efficacy of MnSTF and RT was evaluated in the B16F10‐OVA tumor model (**Figure** **6**a,b). Compared to RT monotherapy, the combination treatment achieved significantly greater tumor growth inhibition (73.1% versus 53.7%) (Figure 6c and Figure S8a, Supporting Information). Importantly, this synergistic effect was not accompanied with overt toxicity, as evidenced by stable body weight throughout the treatment period (Figure S8b, Supporting Information) and the absence of histopathological abnormalities in major organs, including the liver, kidney, and lung (Figure S9, Supporting Information), thereby confirming the favorable biosafety of MnSTF combined with RT. Furthermore, this synergistic effect was further confirmed by the potent antitumor response observed in the poorly immunogenic B16F10 tumor model (Figure S10, Supporting Information).

To elucidate the immune mechanisms underlying the enhanced antitumor efficacy of the combination therapy, key components of innate and adaptive immune activation were analyzed. Elevated intratumoral IFN‐β levels were observed in MnSTF‐treated tumors, irrespective of radiotherapy (Figure 6d). Flow cytometric analysis of tumor‐draining lymph nodes (TDLNs) further revealed a notable increase in the proportion of CD11c^+^ DCs presenting the OVA‐derived SIINFEKL peptide following either RT (1.0 ± 0.4%) or MnSTF (0.9 ± 0.3%) monotherapy. This antigen‐presenting capacity was further enhanced by the combination treatment (1.6 ± 0.2%) relative to either monotherapy (Figure 6e,f; Figure S7c, Supporting Information), indicating a clear synergistic effect. Meanwhile, the MnSTF–RT regimen significantly promoted DC maturation, evidenced by an increased proportion of CD80^+^CD86^+^ DCs in TDLNs (28.6 ± 3.5%) (Figure 6g,h; Figure S7b, Supporting Information), thereby laying the foundation for efficient T cell priming. Building on these results, systemic antigen‐specific T cell responses in the spleen were assessed by IFN‐γ ELISpot assays. Combination therapy induced a 3.4‐fold and 6.2‐fold increase in spot‐forming units compared to MnSTF and RT monotherapy, respectively (Figure 6i,j), demonstrating a pronounced synergistic effect in driving systemic OVA‐specific T cell immunity. Immunofluorescence analysis further demonstrated a significant increase in CD4^+^ and CD8^+^ T cell infiltration within tumor tissues following combination therapy (Figure 6k), indicating successful trafficking of activated effector T cells toward the tumor lesion.

In summary, MnSTF synergizes with radiotherapy by remodeling the local immune microenvironment to enhance antigen presentation and DC maturation, thereby effectively initiating systemic OVA‐specific T cell responses and promoting their efficient mobilization and infiltration into tumor tissues.

### 2.6 MnSTF and mRNA Vaccine Synergy Enhances Antigen Spreading and Antitumor Immunity

**Figure 7:**
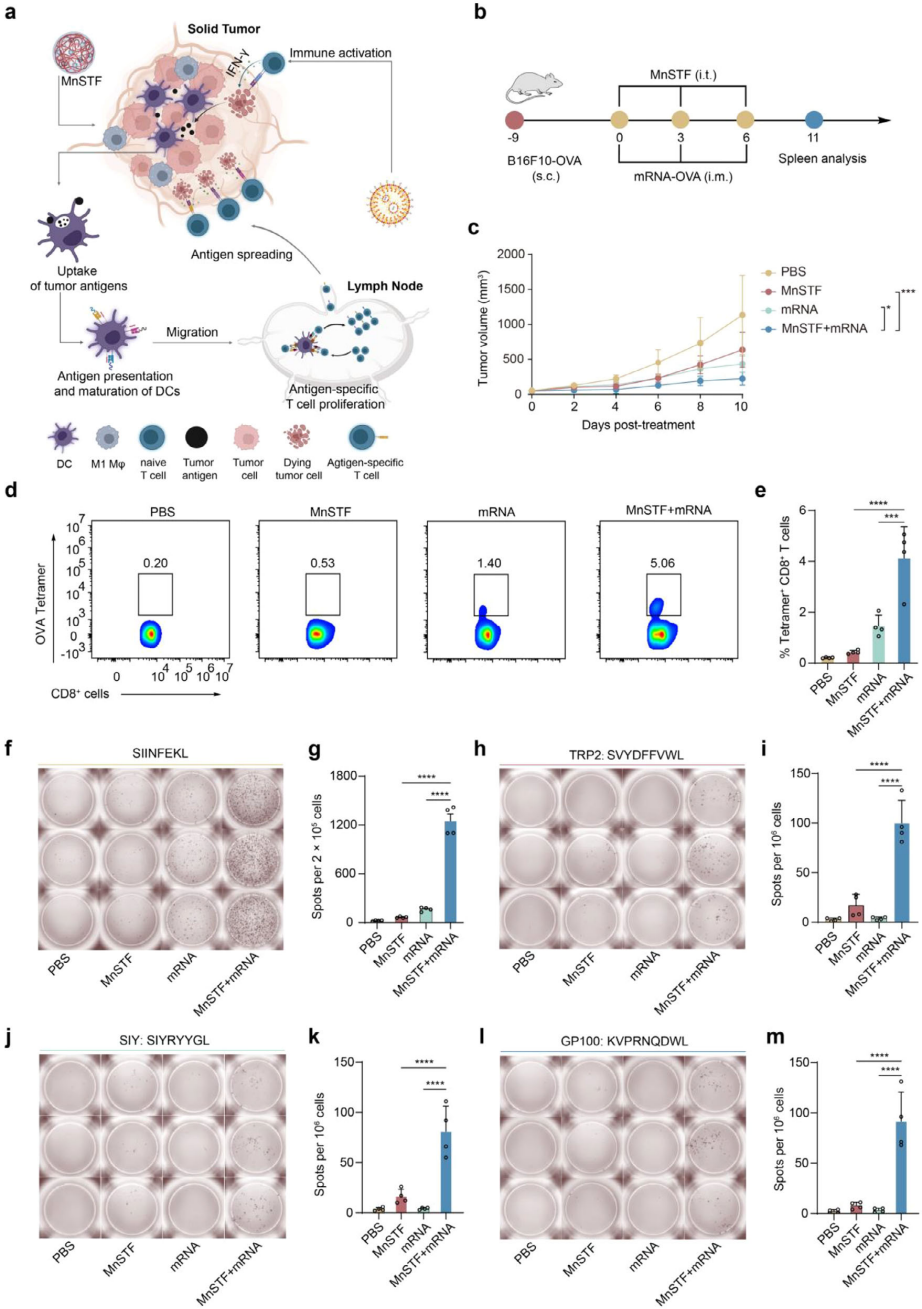
mRNA vaccine and MnSTF synergistically potentiate antitumor immune responses. (a) Schematic illustration of enhanced antitumor immunity induced by mRNA vaccine combined with MnSTF. (b) Experimental design schematic in B16F10‐OVA tumor‐bearing mice treated with PBS, MnSTF (0.69 µmol Mn equivalent), LNP‐formulated mRNA_OVA_ (10 µg), or their combination (MnSTF + mRNA_OVA_). Treatments were administered every three days for three doses. (c) Tumor growth kinetics after indicated treatments (n = 8). (d, e) Representative flow cytometry plots (d) and quantification (e) of SIINFEKL tetramer^+^ CD8^+^ T cells in splenocytes collected at the end of treatment (n = 4). (f, h, j, l) Representative IFN‐γ ELISpot images of splenocytes stimulated with SIINFEKL (f), SVYDFFVWL (h), SIYRYYGL (j), or KVPRNQDWL (l) at treatment end. (g,i,k,m) Quantification of IFN‐γ SFU per 2 × 10^5^ splenocytes for SIINFEKL (g), or per 10^6^ splenocytes for SVYDFFVWL (i), SIYRYYGL (k), and KVPRNQDWL (m), n = 4. Replicates were biological, and the data were expressed as mean ± s.d. Statistical analysis was performed using two‐way ANOVA (c) or one‐way ANOVA (e, g, i, k, m). Significance: *p < 0.05, ***p < 0.001, ****p < 0.0001.

Tumor heterogeneity presents a significant challenge for immunotherapy because diverse antigen expression among tumor cells undermines the efficacy of therapeutic vaccines, resulting in limited coverage, immune evasion, and suboptimal therapeutic outcomes.^[^ ^56^, ^57^, ^58^ ^]^ Therefore, induction of antigen spreading, defined as the stimulation of T cell recognition of tumor epitopes absent from the vaccine, is considered a critical strategy for achieving broad and durable antitumor immunity. However, current mRNA vaccine platforms exhibit limited efficacy in eliciting antigen spreading, leading to suboptimal and poorly polyclonal T cell responses. We thus hypothesize that MnSTF may promote the antigen spreading of mRNA vaccines by reprogramming the tumor microenvironment (**Figure** **7**a).^[^ ^59^ ^]^ In tumor models with limited antigen‐presenting capacity (OVA presentation rate ∼30%), intratumoral administration of MnSTF in combination with mRNA vaccines significantly enhanced antitumor efficacy, yielding a tumor suppression rate of 80.1% (Figure 7b,c; Figure S11, Supporting Information). Compared to mRNA vaccination alone, the combination group exhibited a 2.7‐fold increase in splenic OVA‐tetramer^+^ CD8^+^ T cells (4.1 ± 1.2% versus 1.5 ± 0.4%, Figure 7d,e; Figure S7d, Supporting Information), and a 7.6‐fold increase in IFN‐γ–producing cells as measured by ELISpot (1251 ± 174 versus 164 ± 25 spots per 2 × 10^5^ cells, Figure 7f,g). More importantly, MnSTF markedly promoted antigen spreading, broadening the epitope spectrum targeted by mRNA vaccination (Figure 7h–m). Upon stimulation with endogenous tumor‐associated antigens (TRP2,^[^ ^60^ ^]^ SIY,^[^ ^61^ ^]^ and GP100^[^ ^62^ ^]^) derived from B16F10 cells, splenocytes from the combination group generated substantially higher numbers of IFN‐γ–secreting cells across all tested epitopes (TRP2: 100 ± 23; SIY: 81 ± 25; GP100: 92 ± 29 spots per 10^6^ cells), whereas the mRNA‐only group did not differ significantly from PBS controls. These results indicate that MnSTF not only enhances the immune recognition and response to the initial antigen but also effectively overcomes vaccine‐induced antigenic limitation, promoting immune recognition of non‐targeted epitopes and thereby significantly expanding the immunological coverage and therapeutic potential of mRNA vaccines against tumor heterogeneity.

### 2.7 MnSTF Adjuvant Synergizes with Tumor‐Associated Antigens to Enhance Vaccine Efficacy

**Figure 8:**
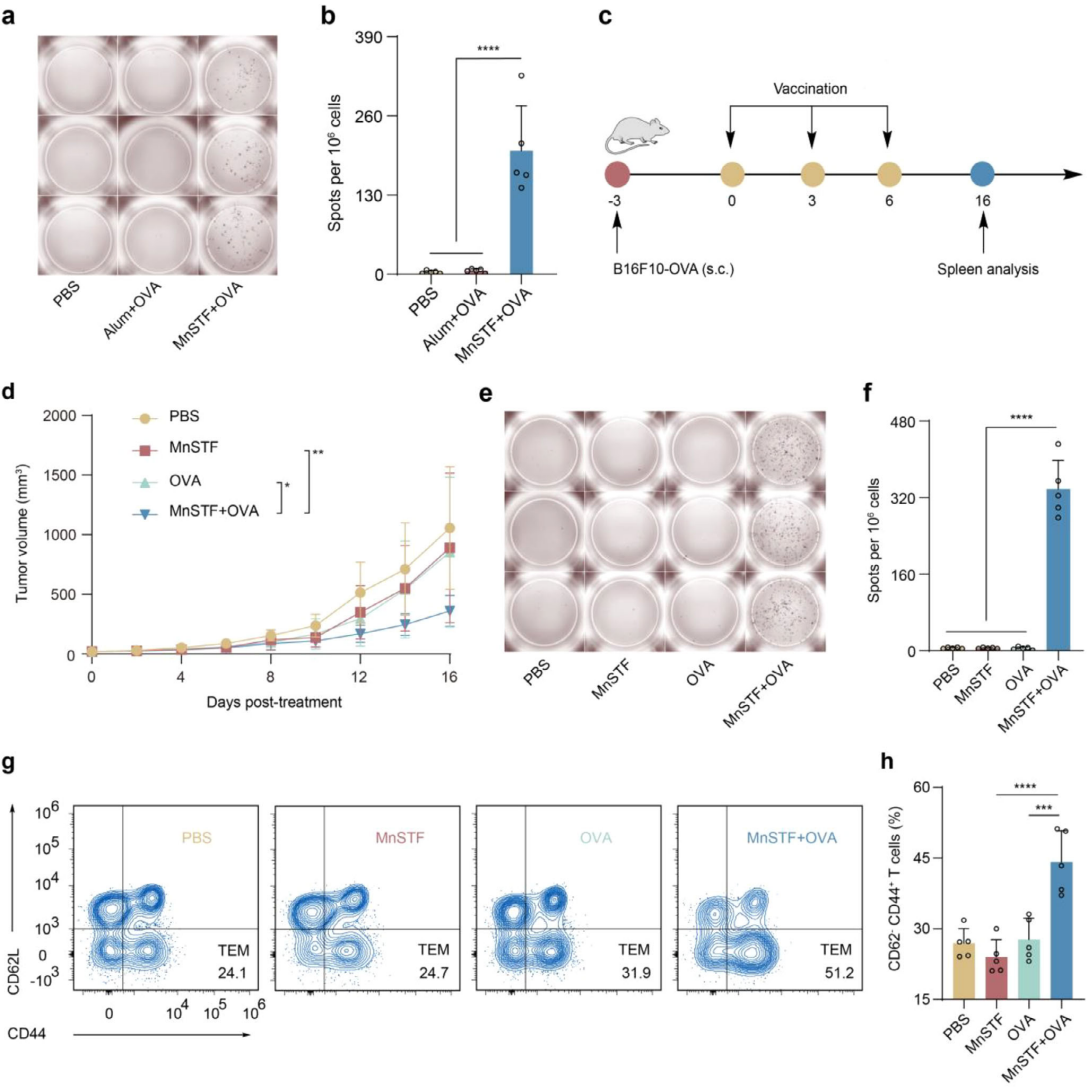
MnSTF acts as a vaccine adjuvant boosting tumor antigen‐specific immunity. (a, b) C57BL/6 mice were subcutaneously immunized with PBS, Alum + OVA (50 µg Alum; 100 µg OVA), or MnSTF + OVA (50 µg Mn; 100 µg OVA) every three days for a total of three doses. Spleens were harvested 7 days after the final immunization to quantify OVA‐specific IFN‐γ–producing splenocytes by ELISpot. Representative IFN‐γ ELISpot images (a) and quantification of SFU per 10^6^ splenocytes (b). (c) Schematic of the experimental design in B16F10‐OVA tumor‐bearing mice treated subcutaneously with PBS, MnSTF (0.69 µmol Mn equivalent), OVA (100 µg), or their combination (MnSTF + OVA). Treatments were administered every three days for three doses. (d) Tumor growth kinetics following the indicated treatments (n = 8). (e, f) Representative IFN‐γ ELISpot images (e) and quantification of SFU per 10^6^ splenocytes (f), n = 5. (g, h) Representative flow cytometry plots (g) and quantification (h) of effector memory CD44^+^CD62L−CD8^+^ T cells (n = 5). Replicates were biological, and the data were expressed as mean ± s.d. Statistical analysis was performed using two‐way ANOVA (d) or one‐way ANOVA (b, f, h). Statistical significance: *p < 0.05, **p< 0.01, ***p< 0.001, ****p< 0.0001.

The clinical utility of conventional adjuvants in cancer vaccines is fundamentally constrained by their inherent bias toward humoral immunity.^[^ ^63^ ^]^ Prototypical agents such as aluminum salts can induce robust antibody responses but fail to effectively activate cellular immune pathways—particularly cytotoxic CD8^+^ T cell responses critical for tumor clearance.^[^ ^64^ ^]^ This immunological imbalance poses a major barrier to their application of conventional adjuvants in therapeutic cancer vaccination. To evaluate the immunostimulatory capacity of the nano‐adjuvant MnSTF versus a conventional aluminum adjuvant in eliciting cellular immunity, C57BL/6 mice were subcutaneously immunized with ovalbumin (OVA) formulated with each adjuvant. Seven days after the final immunization, splenocytes were harvested and analyzed by IFN‐γ ELISpot assay. The MnSTF‐adjuvanted group elicited a robust antigen‐specific T cell response, achieving 203 ± 74 SFU per 10^6^ splenocytes, significantly higher than those induced by the aluminum‐adjuvanted and PBS control groups. No statistically significant difference was observed between the aluminum and PBS groups (**Figure** **8**a,b).

To further validate the therapeutic potential of MnSTF as a vaccine adjuvant, its efficacy was assessed in a B16F10‐OVA tumor‐bearing C57BL/6 mouse model (Figure 8c). Compared with either OVA antigen or MnSTF alone, the combination treatment resulted in significantly enhanced tumor suppression, achieving an inhibition rate of 65.8% (Figure 8d; Figure S12, Supporting Information), indicating that MnSTF could effectively translate its adjuvant function into therapeutic antitumor effects. At the immunological endpoint, ELISpot analysis showed a marked increase in IFN‐γ–secreting splenocytes in the combination group, averaging 338 ± 60 spots per 10^6^ cells, which was significantly higher than that observed in either monotherapy group (Figure 8e,f), highlighting the potent capacity of MnSTF to augment antigen‐specific cellular immune responses in vivo. Beyond the initial immune activation, MnSTF's capacity to induce immunological memory was further assessed. Flow cytometry revealed that OVA + MnSTF immunization significantly increased the proportion of splenic CD8^+^CD44^+^CD62L^−^ effector memory T cells (TEM) to 44.1 ± 6.7%, substantially higher than those observed in either OVA or MnSTF monotherapy groups (Figure 8g,h and Figure S7e, Supporting Information). These findings indicate that MnSTF not only elicits potent primary T cell responses but also promotes long‐term memory immunity, which is critical for preventing tumor relapse and immune evasion in solid malignancies.

## 3 Conclusion

In this study, we devised a novel self‐assembled nanoimmunomodulator, MnSTF, based on the “soft‐drug” design concept, to synergistically potentiate cGAS‐STING pathway activation via local administration (e.g., intratumoral or subcutaneous injection). Following potent local immune activation, the MnSTF complex gradually dissociates, enters systemic circulation, and its functional components lose their synergistic properties, thereby attenuating the immune coupling effect and substantially reducing systemic exposure and toxicity risks. Safety assessments demonstrated that MnSTF did not trigger cytokine storm or acute liver injury under either therapeutic or supratherapeutic‐dose regimens, indicating favorable tolerability and biocompatibility. Mechanistic studies further confirmed that intratumoral administration of MnSTF significantly upregulates local type I interferon (IFN‐I) production and promotes recruitment and activation of CD11c^+^ dendritic cells and iNOS⁺ M1 macrophages, thereby orchestrating the reshaping of the tumor immune microenvironment. In combination with radiotherapy, MnSTF markedly enhanced local immune activation, elicited systemic antitumor T cell responses, and facilitated robust infiltration of CD8^+^ T cells into tumor tissue. Moreover, in the synergistic application with mRNA vaccines, MnSTF not only enhanced antigen‐specific T cell responses to mRNA‐encoded epitopes but also promoted epitope spreading, stimulated polyclonal, broad‐spectrum T cell immunity, thereby expanding the vaccine's immunological coverage of tumor heterogeneity. As an adjuvant for therapeutic tumor vaccines, MnSTF potently induces antigen‐specific CD8^+^ T cell responses when combined with OVA subunit vaccine and significantly increases the proportion of CD44^+^CD62L^−^ effector memory T cells (TEM), highlighting robust immune persistence and long‐term protective potential. Therefore, as a “soft‐drug” nanoadjuvant, MnSTF demonstrates potent cellular immune activation and memory induction capabilities in both in situ and conventional vaccine models, offering novel strategies and technical support for the development of efficient and safe tumor vaccine adjuvants.

## 4 Experimental Section

### Formation of MnSTF

STF‐1623 (30 mM) was dissolved in 10 mL of deionized water, adjusted to pH 7.4 using 1 N NaOH, and sterile‐filtered through a 0.22 µm filter. Similarly, manganese (II) chloride tetrahydrate (MnCl_2_·4H_2_O, 30 mM) was dissolved in 10 mL of deionized water and sterile‐filtered (0.22 µm). The STF‐1623 solution was added dropwise to the MnCl_2_ solution to facilitate self‐assembly. After 20 minutes, the crude MnSTF was purified by centrifugation (4000 rpm, 10 min, 4 °C) and washed twice with PBS (pH 7.4, 0.01 M) to remove unreacted precursors and stabilize the nanoparticles. Finally, MnSTF was resuspended in PBS and sonicated on ice to disperse into nanoparticles using an ultrasonic cell disruptor (450 W, 55% amplitude, 10 min, pulse mode: 5 s on/2 s off). The preparation of ICG–MnSTF followed the same procedure as MnSTF. Briefly, the mixture solution of STF‐1623 (30 mM, pH 7.4) and indocyanine green (ICG, 0.3 mM, pH 7.4) was added dropwise to MnCl_2_ solution (30 mM), the subsequent steps were the same as those for MnSTF.

### Characterization of MnSTF

The morphologies, crystal structures, and elemental compositions were characterized by transmission electron microscopy (TEM) (Hitachi HT7700, Japan) and high‐resolution TEM (HRTEM) combined with energy‐dispersive X‐ray spectroscopy (EDS) (JEM‐2100F, JEOL, Japan). High‐angle annular dark‐field scanning transmission electron microscopy (HAADF‐STEM) images were acquired using a double Cs‐corrected transmission electron microscope (Thermo Scientific Spectra 300) operated at 300 kV with a beam current of 10–20 pA. Powder X‐ray diffraction (PXRD) patterns were obtained by a D8 ADVANCE diffractometer (Bruker, Germany) with Cu Kα radiation (λ = 1.54 Å). X‐ray photoelectron spectroscopy (XPS) spectra were measured by a PHI 5000 VersaProbe (ULVAC‐PHI, Japan) with Al Kα radiation (hν = 1486.6 eV). UV–vis absorption spectra were recorded with a Shimadzu UV–vis spectrophotometer (UV3600, Japan). Fourier transform infrared (FTIR) spectra were recorded with a FTIR spectrometer (Bruker, Vertex 80v and Tensor 27, Germany). The size and surface charge of the nanoparticles were determined by dynamic light scattering using a Zetasizer Nano ZS equipped with a He–Ne laser (λ = 633 nm) at a scattering angle of 137° (Malvern Instruments).

### Quantification of MnSTF

Before content determination, formic acid was added to the MnSTF nanoparticle solution to acidify it into a clear solution. The content of Mn^2+^ was determined using the formaldoxime colorimetric method by measuring the absorbance at 450 nm. STF‐1623 was quantified by reversed‐phase high‐performance liquid chromatography (RP‐HPLC) at ambient temperature using a C18 chromatography column (Morphling, WD‐C18, 4.6 × 150 mm, 5 µm). The flow rate was 1.0 mL/min, and the detection wavelength was 320 nm. The HPLC mobile phases consisted of (A) water with 0.1% formic acid and (B) acetonitrile with 0.1% formic acid (FA). The gradient elution profile was as follows: 0–10 min, linear from 5% to 50% B; 10–12 min, held at 50% B; 12–15 min, linear from 50% to 5% B; 15–20 min, maintained at 5% B to re‐equilibrate the column.

### In Vitro Drug Release Studies

To study drug release at different pH values, free MnCl_2_ (800 µL, 20 mM), free STF‐1623 (800 µL, 40 mM) and MnSTF (800 µL, 20 mM Mn^2+^/40 mM STF‐1623) were placed in dialysis bags (Solarbio; molecular weight cutoff: 10 kDa) individually and immersed in 45 mL of Tris‐HCl buffer (pH 7.4 or 6.5). The samples were incubated at 37 °C with shaking (120 rpm), and at predetermined time points, 2 mL of the release medium was withdrawn and replaced with an equal volume of fresh buffer. The concentrations of Mn^2+^ and STF‐1623 were measured using the formaldoxime colorimetric method and RP‐HPLC, respectively.

### Preparation of mRNA‐OVA

The plasmid contained the T7 promoter sequence 5′‐TAATACGACTCACTATAAG‐3′. After specific enzymatic digestion for linearization, it was purified using a gel extraction kit (Omega, USA). In vitro transcription (IVT) of mRNA was carried out using a T7 RNA polymerase transcription kit (Jiangsu Shenji Biotechnology, China). The transcription reaction system included: purified DNA template, ATP, CTP, GTP, N1‐Me‐pUTP, as well as CAP GAG and T7 RNA polymerase mix. After incubating the reaction at 37 °C for 2 h, DNase I was added to remove the template DNA. The transcription product was purified by lithium chloride precipitation and then resuspended in nuclease‐free water. Subsequently, the concentration of mRNA was determined using a NanoDrop micro‐spectrophotometer (Thermo Fisher Scientific, USA). The lipid components of LNP included SM‐102, DSPC, cholesterol, and DMG‐PEG2000, with a molar ratio of 50:10:38.5:1.5. The lipid components were dissolved in absolute ethanol at a total lipid concentration of 12 mM, while the mRNA was dissolved in 50 mM citrate buffer (pH 4.0). mRNA‐LNP was formulated by microfluidic mixing at a 3:1 volume ratio (aqueous: organic phase) using a microfluidic device (FluidicLab, China). The obtained LNP was dialyzed against phosphate‐buffered saline (PBS, pH 7.4) through a 10 kDa molecular weight cut‐off membrane (Thermo Fisher Scientific, USA) to remove ethanol and adjust the pH. Finally, the formulation was sterile‐filtered through a 0.22 µm membrane. The encapsulation efficiency of mRNA was determined using the RiboGreen RNA detection kit (Thermo Fisher Scientific, USA).

### Cell Lines

The B16F10‐OVA (RRID:CVCL_WM78), RAW264.7 (RRID:CVCL_0493), and B16F10 (RRID:CVCL_0159) cell lines were obtained from the American Type Culture Collection (ATCC, Manassas, VA, USA) in March 2021, July 2022, and August 2022, respectively, where they were authenticated by morphology, karyotyping, and PCR‐based approaches. B16F10‐OVA and B16F10 cells were cultured in RPMI 1640 medium supplemented with 10% fetal bovine serum (FBS), 100 U/mL penicillin, 100 µg/mL streptomycin, and 50 µg/mL gentamicin. RAW264.7 cells were maintained in DMEM supplemented with 10% heat‐inactivated FBS (heated at 58 °C for 30 min), 100 U/mL penicillin, 100 µg/mL streptomycin, and 50 µg/mL gentamicin. The DC2.4 (RRID:CVCL_J409) cell line was purchased from FuHeng Biology (Shanghai, China) in April 2024 and maintained in RPMI 1640 medium supplemented with 10% FBS, 100 U/mL penicillin, 100 µg/mL streptomycin, 50 µg/mL gentamicin, and 10 ng/mL granulocyte‐macrophage colony‐stimulating factor (GM‐CSF). All cell lines were maintained in a humidified incubator at 37 °C with 5% CO_2_ and were tested and confirmed to be free of mycoplasma contamination.

### Animal

C57BL/6 mice (5 weeks old) were obtained from Yangzhou University Medical Center (Yangzhou, China). All mice were housed under specific pathogen‐free (SPF) conditions with free access to standard chow and water, maintained on a 12‐h light/dark cycle at 22 °C and 40–70% relative humidity. All animal procedures were approved by the Institutional Animal Care and Use Committee of Nanjing University (IACUC‐D2202160) and conducted in accordance with the guidelines of the Association for Assessment and Accreditation of Laboratory Animal Care International (AAALAC) and all applicable national and institutional regulations.

### Cell Viability

A Cell Counting Kit‐8 (CCK‐8) assay was performed to assess the biocompatibility of MnSTF. Briefly, RAW264.7 and DC2.4 cells were seeded in a 96‐well plate (5 × 10^4^ cells/well) and allowed to adhere for 24 h. The medium was then aspirated and replaced with fresh medium containing serial concentrations of MnSTF. Following 24 h of treatment, the treatment medium was removed, and cells were incubated with CCK‐8 solution (10% v/v in fresh culture medium) for 0.5 h. The absorbance was measured at 450 nm using a microplate reader. Relative cell viability was expressed as a percentage of the untreated control, calculated using the following formula: Cell viability (%) = (mean absorbance value of treatment group/mean absorbance value of control) × 100%.

### In Vitro Evaluation STING Pathway Activation—RT–PCR Analysis

RAW264.7 cells were seeded in a six‐well plate at a density of 1.5 × 10^6^ cells per well. After cell attachment, the cells were treated with 0.3 mM Mn^2+^, 0.6 mM STF‐1623, or 0.3 mM MnSTF (calculated based on Mn concentration) for the indicated time periods. Total RNA was extracted with AFTSpin Tissue/Cell Fast RNA Extraction Kit (ABclonal, Cat. RK30120). Complementary DNA (cDNA) was synthesized with ABScript Neo RT Master Mix for qPCR with gDNA Remover (ABclonal, Cat. RK20432). Real‐time quantitative polymerase chain reaction (RT‐qPCR) was performed using the Taq Pro Universal SYBR qPCR Master Mix (Vazyme, Cat. Q712‐02) on an ABI QuantStudio 5 system (Thermo Fisher Scientific). The use of the above kits followed the manufacturer's instructions. Data were analyzed using QuantStudio Design & Analysis Software (v1.5.2). Gene expression levels were normalized to GAPDH, and fold changes relative to the PBS group were calculated using the 2^−ΔΔCt^ Method. The sequences of qPCR primers were listed as follows:

Mouse GAPDH (forward): 5′‐TGACGTGCCGCCTGGAGAAA‐3′

Mouse GAPDH (reverse): 5′‐AGTGTAGCCCAAGATGCCCTTCAG‐3′

Mouse IFN‐β1 (forward): 5′‐TCCAGCTCCAAGAAAGGACG‐3′

Mouse IFN‐β1 (reverse): 5′‐TTGAAGTCCGCCCTGTAGGT‐3′

### Western Blot Analysis

The RAW264.7 cells were treated as described above, and exposure time was 6 h. The treated RAW264.7 cells were collected and washed with cold PBS and lysed with RIPA lysis buffer (Servicebio, Cat. G2002) supplemented with protease inhibitor cocktail (Servicebio, Cat. G2008) and phosphatase inhibitors (Servicebio, Cat. G2007) on ice for 10 min. Lysates were centrifuged at 12 000 × *g* at 4 °C for 10 min, and the supernatants were collected. Total protein concentration was determined using a bicinchoninic acid (BCA) protein assay kit (Beyotime, Cat. P0009). Equal amounts of protein were mixed with 5× SDS loading buffer and heated at 70 °C for 10 min, and separated by 12% SDS–PAGE at 120 V for approximately 60 min. Proteins were transferred onto polyvinylidene fluoride (PVDF) membranes at 200 mA for 90 min. The membranes were washed with 1× TBST (Tris‐buffered saline containing 0.1% Tween‐20), blocked with 5% (w/v) BSA at 4 °C overnight, and incubated with the following primary antibodies (all from Cell Signaling Technology, 1:1000 dilution): cGAS (D1D3G) Rabbit mAb (Cat. 15 102), Phospho‐STING (Ser366) (D7C3S) Rabbit mAb (Cat. 19 781), STING (D2P2F) Rabbit mAb (Cat. 13 647), Phospho‐TBK1/NAK (Ser172) (D52C2) XP Rabbit mAb (Cat. 5483), TBK1/NAK (D1B4) Rabbit mAb (Cat. 3504), Phospho‐IRF‐3 (Ser396) (4D4G) Rabbit mAb (Cat. 4947), IRF‐3 (D83B9) Rabbit mAb (Cat. 4302), Phospho‐NF‐κB p65 (Ser536) (93H1) Rabbit mAb (Cat. 3033), NF‐κB p65 (D14E12) XP Rabbit mAb (Cat. 8242), GAPDH (14C10) Rabbit mAb (Cat. 2118). After washing three times with 1× TBST, PVDF membranes were incubated with an HRP‐conjugated secondary antibody (anti‐rabbit IgG, HRP‐linked antibody, Cat. 7074) for 60 min at room temperature. Protein bands were visualized using enhanced chemiluminescence (ECL) substrate and imaged with a Tanon 5200 system.

### Detection of 2′,3′‐cGAMP and Cytokines, and Dendritic Cell Maturation Assay

The cells were treated as described above with an exposure time of 24 h. The culture supernatant from RAW264.7 cells or DC2.4 was collected for ELISA assays to quantify 2′,3′‐cGAMP (COIBO BIO, Cat. CB15107‐Mu), IFN‐β (ABclonal, Cat. RK00420), TNF‐α (ABclonal, Cat. RK00027), and IL‐6 (Dakewe, Cat. 1 210 602)]. All the ELISA kits were used according to the manufacturer's instructions. The treated DC2.4 cells were first incubated with anti‐CD16/32 antibody (BioLegend, Cat. 156 603) for Fc receptor blocking, followed by staining with APC anti‐mouse CD80 antibody (BioLegend, Cat. 104 714) or PE anti‐mouse I‐A/I‐E antibody (BioLegend, Cat. 107 607) at 4 °C for 1 h. After being washed with PBS, the cells were analyzed using a Beckman Coulter CytoFLEX S flow cytometer. Data were analysed in FlowJo v10.8.1 or CytExpert v2.4.

### In Vivo Imaging System Imaging

Male C57BL/6 mice were subcutaneously inoculated with 5 × 10^5^ B16F10‐OVA cells on the right flank. When the tumor volume reached approximately 80 mm^3^, tumors were intratumorally injected with either 12.5 µg of free ICG or 12.5 µg of ICG‐labeled MnSTF. Mice were anesthetized via intraperitoneal injection of pentobarbital (0.1 mg per mouse). After treatment, the mice were imaged using the IVIS Spectrum imaging system (Tanon ABL‐X5) under epi‐illumination fluorescence settings with a 0.5‐second exposure time. Image analysis was performed using Living Image software (Tanon), and data were normalized to the initial radiance for each mouse.

### In Vivo Cytokine Detection

For IL‐6 detection: Briefly, the blood was collected into anticoagulant tube from the orbital cavity of mice at the indicated time points after subcutaneous administration of different doses of MSA‐2 or MnSTF, as previously described. Then, the samples were centrifuged at 2000 ×g for 10 min at 4 °C, and the plasma was used for IL‐6 detection (Dakewe, Cat. 1 210 602). For IFN‐β detection (MULTI SCIENCES, Cat. EK2236), tumor tissues were harvested one day after treatment with PBS, MnSTF, RT, or their combination (RT + MnSTF) in B16F10‐OVA tumor‐bearing mice. Tissues were minced in ice‐cold PBS containing 1% PMSF and subsequently homogenized using an ultrasonic cell disruptor (450 W, 25% amplitude, pulse mode: 2 s on / 3 s off). The homogenates were then centrifuged at 12 000 rpm for 10 min at 4 °C, and the supernatant was collected for IFN‐β quantification.

### IFN‐γ ELISpot

In the B16F10‐OVA tumor‐bearing mouse model, IFN‐γ ELISpot assays were conducted after treatment using a mouse IFN‐γ precoated ELISpot kit (Dakewe, Cat. 2 210 005). The mice were euthanized, and spleens were harvested to prepare single‐cell suspensions. The cells were resuspended in serum‐free RPMI 1640 medium and seeded into an ELISpot plate at a specified density per well. The splenocytes were then stimulated with the following peptides: SIINFEKL (1 µg/mL), SVYDFFVWL (2 µg/mL), SIYRYYGL (2 µg/mL), and KVPRNQDWL (2 µg/mL). Ionomycin and phorbol 12‐myristate 13‐acetate (PMA) were used as positive controls. After incubation for 18 h at 37 °C with 5% CO_2_, the IFN‐γ ELISpot assay was completed according to the manufacturer's instructions. Spots were quantified using a Mabtech IRIS FluoroSpot/ELISpot reader (Mabtech, Sweden).

### In Vivo Flow Cytometry

For the analysis of immune cells in tumors, lymph nodes, and spleens, samples were collected at the indicated time points following various treatments. On day 1 post‐treatment, tumor‐draining lymph nodes were collected, gently dissociated, and filtered through a 70 µm cell strainer to obtain single‐cell suspensions. To assess DC maturation, cells were first blocked with anti‐mouse CD16/32 antibody, followed by staining with FITC anti‐mouse CD11c antibody (BioLegend, Cat. 117 306), APC anti‐mouse CD80 antibody (BioLegend, Cat. 104 714), and PE anti‐mouse CD86 antibody (BioLegend, Cat. 105 008). For the evaluation of antigen presentation by DCs, cells were stained with FITC anti‐mouse CD11c antibody and PE anti‐mouse H‐2K^b^ bound to SIINFEKL antibody (BioLegend, Cat. 141 604). For tumor samples, tumors were excised, minced, and digested with 1 mg/mL collagenase II (YEASEN, Cat. 40508ES60) and 0.1 mg/mL DNase I (YEASEN, Cat. 10608ES60) at 37 °C for 60 min with gentle shaking. The resulting suspensions were filtered through a 70 µm strainer, washed with flow cytometry staining buffer (Proteintech, Cat. PF00018), and lysed using ACK lysing buffer (YEASEN, Cat. 40401ES60) on ice for 4 minutes. After Fc blocking, cells were stained for macrophage analysis with FITC anti‐mouse F4/80 antibody (BioLegend, Cat. 123 108) and PE anti‐Nos2 (iNOS) antibody (BioLegend, Cat. 696 805). As iNOS is an intracellular marker of macrophages, cells were fixed and permeabilized using the BD Cytofix/Cytoperm Fixation/Permeabilization Kit (BD Biosciences, Cat. 554 714). For the analysis of M1/M2 macrophages and regulatory T cells (Tregs) in tumor tissue, 3 days after a single intratumoral administration, the cells processed as described above were stained with the following antibody panels: (1) APC/Cyanine7 anti‐mouse CD45 antibody (BioLegend, Cat. 103 115), PE anti‐mouse F4/80 antibody (BioLegend, Cat. 123 109), Brilliant Violet 421 anti‐mouse CD86 antibody (BioLegend, Cat. 105 032), and APC anti‐mouse CD206 (MMR) antibody (BioLegend, Cat. 141 708); and (2) APC/Cy7‐anti‐CD45, APC anti‐mouse CD3 antibody (BioLegend, Cat. 100 236), PE anti‐mouse CD4 antibody (BioLegend, Cat. 100 408), and Brilliant Violet 421 anti‐mouse FOXP3 antibody (BioLegend, Cat. 126 419). As FOXP3 is a nuclear transcription factor, intracellular staining for FOXP3 was performed using the True‐Nuclear Transcription Factor Buffer Set (BioLegend, Cat. 424 401). For general antigen‐presenting cell (APC) analysis, cells were stained with FITC anti‐mouse CD11c antibody. To quantify tetramer⁺ CD8⁺ T cells, spleens were collected on day 5 post‐final treatment. Single‐cell suspensions were prepared, lysed with ACK buffer, and stained with FITC anti‐mouse CD8α (BioLegend, Cat. 100 804) and PE‐labeled H‐2K^b^/OVA (SIINFEKL) MHC Tetramer (HELIXGEN, Cat. HG08T14028). For central/effector memory T cells (TCM/TEM), splenocytes were collected on day 10 post‐final treatment and stained with FITC anti‐mouse CD8α antibody, APC anti‐mouse/human CD44 antibody (BioLegend, Cat. 103 012), and Brilliant Violet 421 anti‐mouse CD62L antibody (BioLegend, Cat. 104 435). All samples were analyzed using a Beckman Coulter CytoFLEX S flow cytometer, and data were processed using FlowJo v10.8.1.

### Tumor Immunotherapy

To evaluate the therapeutic effects, 5 × 10^5^ B16F10‐OVA or B16F10 cells were injected subcutaneously into the right flanks of male C57BL/6 mice. When tumors reached approximately 70 mm^3^, mice were randomly assigned to various treatment groups. Treatments were administered as follows: PBS (i.t., 25 µL), MSA‐2 (i.t., 0.41 µmol in 25 µL), MnSTF (i.t., 0.69 µmol Mn equivalent in 25 µL), and radiation (6 Gy). For MnSTF combined with mRNA‐OVA therapy, 5 × 10^5^ B16F10‐OVA cells were injected subcutaneously into the right flanks of male C57BL/6 mice. On day 9 post‐inoculation, tumor‐bearing mice were randomly assigned to four experimental cohorts using block randomization. The mice were treated with PBS (i.t., 25 µL), MnSTF (i.t., 0.69 µmol Mn equivalent in 25 µL), mRNA (10 µg in 100 µL), or their combination. For MnSTF combined with OVA antigen, 5 × 10^5^ B16F10‐OVA cells were injected subcutaneously into the right flanks of male C57BL/6 mice. On day 3 post‐inoculation, tumor‐bearing mice were randomly assigned to four experimental cohorts using block randomization. The mice were treated with PBS (s.c., 100 µL), MnSTF (s.c., 0.69 µmol Mn equivalent in 100 µL), OVA (100 µg in 100 µL), or their combination (100 µL).

Tumor volumes were measured using calipers and calculated using the formula V = (width^2^ × length) / 2. Mice were euthanized when the tumors reached a volume of 2000 mm^3^ or exhibited signs of poor body condition. At the end of the experimental period, the tumor inhibition rate (TIR) was calculated as follows: TIR (%) = [(mean tumor volume of control group − mean tumor volume of treatment group) / mean tumor volume of control group] × 100.

### Transcriptome Sequencing Analysis (RNA‐seq)

Tumor tissues were excised on days 1, 3, and 5 after PBS or MnSTF treatment and sent to Beijing Biomarker Technologies Co., Ltd. for further sample processing and RNA sequencing. RNA extraction, library construction, and sequencing: Total RNA was extracted from tumor tissues using TRIzol Reagent (Life Technologies, California, USA) according to the manufacturer's instructions. RNA integrity and concentration were assessed using the Agilent 2100 Bioanalyzer (Agilent Technologies, Inc., Santa Clara, CA, USA). mRNA was isolated by NEBNext Poly (A) mRNA Magnetic Isolation Module (NEB, E7490). The cDNA library was constructed using the NEBNext Ultra RNA Library Prep Kit for Illumina (NEB, E7530) and NEBNext Multiplex Oligos for Illumina (NEB, E7500), following the manufacturer's protocols. In brief, the enriched mRNA was fragmented into ∼200 nt RNA inserts and used for first and second‐strand cDNA synthesis. The resulting double‐stranded cDNA was subjected to end‐repair, A‐tailing, and adaptor ligation. The suitable fragments were isolated by Agencourt AMPure XP beads (Beckman Coulter, Inc.) and enriched by PCR amplification. Final libraries were sequenced on an Illumina HiSeq platform.

Transcriptome analysis using reference genome‐based reads mapping: Low‐quality reads were removed using a Perl script. These included adapter‐only reads, reads containing more than 5% ambiguous bases (N), or reads with less than 20% of bases having a Phred quality score of Q20 or above (i.e., a sequencing error rate below 1%). The filtered clean reads were then mapped to the *Mus musculus* reference genome (GRCm39) using TopHat2 (Kim et al., 2013). The aligned BAM/SAM files were further processed to remove potential PCR duplicates. Gene expression levels were quantified as FPKM (fragments per kilobase of transcript per million mapped reads) using Cufflinks (Trapnell et al., 2010).

Identification of Differential Gene Expression: Differentially expressed genes between experimental groups were identified using DESeq (Anders and Huber, 2010), and Q‐values were calculated to assess statistical significance. Gene expression differences were quantified based on the ratio of FPKM values between samples. The false discovery rate (FDR) method was applied to adjust *P*‐values for multiple testing. Genes with an absolute log_2_ fold change ≥ 2 and FDR < 0.01 were considered significantly differentially expressed and included in subsequent analyses.

Sequence Annotation: Transcript sequences from mouse tumor tissues were annotated by comparison against several databases. Protein‐coding genes were identified using BLASTX searches against the NCBI non‐redundant protein (Nr) and Swiss‐Prot databases with an E‐value cutoff of 1e‐5. Additionally, BLASTn was performed against the NCBI non‐redundant nucleotide (Nt) database using the same cutoff value. Functional annotations were assigned based on the best BLAST hits (i.e., those with the highest alignment scores). For Gene Ontology (GO) annotation, BLAST results were imported into Blast2GO (Conesa et al., 2005), and GO terms were assigned accordingly. Annotated genes were mapped to GO terms across the three GO categories: biological process, cellular component, and molecular function. GO functional classifications were visualized using in‐house Perl scripts, and GO term enrichment analysis was performed using the TopGO package in R. Genes were also aligned to the Clusters of Orthologous Groups (COG) database for functional classification (Tatusov et al., 2000). Kyoto Encyclopedia of Genes and Genomes (KEGG) pathway annotations were assigned using in‐house Perl scripts, enabling the identification of enriched signaling pathways and biological processes relevant to tumor progression and immune responses in mice.

### Proteomics

After subcutaneous injection of PBS (100 µL), MnSTF (100 µL, 0.69 µmol Mn equivalent), or MSA‐2 (100 µL, 0.41 µmol) into mice, blood was collected from the orbital sinus four hours post‐injection. Plasma was then sent to Shanghai Biotree Biomedical Technology Co., Ltd. for further sample processing and data acquisition. Sample preparation was performed in three sequential steps: protein extraction, enzymatic digestion, and peptide desalting. nanoLC­MS/MS analysis: for each sample, 500 ng of total peptides were separated and analyzed with a nano­UPLC (Vanquish neo) coupled to an Astral instrument (Thermo Scientific) with a nano­electrospray ion source. Separation was performed using a reversed­phase column (EASY‐Spray HPLC (150 µm× 15 cm), Thermo Scientific, USA). Mobile phases were H_2_O with 0.1% FA (phase A) and 80% ACN with 0.1% FA (phase B). Sample separation was performed using an 8‐min gradient. Data independent acquisition (DIA) was performed in profile and positive mode with Orbitrap analyzer at a resolution of 240K and m/z range of 380­980 for MS1; for MS2 m/z range of 150­2000. The HCD was performed with a normalized collision energy (NCE) of 25% and an isolation window of 2 m/z. DIA‐NN database search: Vendor's raw MS files were processed using DIA‐NN software (1.8.1). MS spectra lists were searched against the UniProt *Mus musculus* reference proteome (uniprot_Mus_musculus_10 090_reviewed_2024.fasta), Carbamidomethyl [C] as a fixed modification, Oxidation (M), and Acetyl (Protein N­term) as variable modifications. Trypsin was used as a protease. A maximum of two missed cleavage(s) was allowed. The false discovery rate (FDR) was set to 0.01 for both PSM and peptide levels. Peptide identification was performed with an initial precursor mass deviation of up to 20 ppm and a fragment mass deviation of 20 ppm. All the other parameters were reserved as default.

### Immunofluorescence Staining for CD4⁺ and CD8⁺ T Cell Infiltration

For tumor section analysis, tumor tissues from each group were harvested after treatment completion and fixed in 4% paraformaldehyde overnight. Samples were then sent to Wuhan Servicebio Technology Co., Ltd. for immunofluorescence (IF) analysis.

In brief, after deparaffinization and antigen retrieval, frozen tumor sections were blocked with 5% BSA solution for 1 h at room temperature, followed by overnight incubation at 4 °C in a humidified chamber with primary antibodies against CD4 and CD8 (Abcam, USA) for T‐cell analysis. After washing the sections three times with 1× PBS containing 0.1% Tween‐20 (PBST), the sections were incubated with fluorophore‐conjugated secondary antibodies for 1 hour at room temperature in the dark. To remove unbound antibodies, the sections were washed three times with 1× PBST (5 min each). Nuclei were counterstained with 4′,6‐diamidino‐2‐phenylindole (DAPI). Fluorescent images were acquired using a confocal fluorescence microscope (Nikon, Japan) and analyzed with ImageJ software.

### Safety Evaluation

For hematological and biochemical assessment, body weight (W_0_) of male C57BL/6 mice was measured prior to subcutaneous injection of PBS, MSA‐2, or MnSTF. At 24 h post‐treatment, body weight (W_1_) was measured again, and whole blood collected in EDTA‐coated tubes was used for hematological analysis. Serum was obtained by centrifuging the blood at 2000 × g for 10 min at 4 °C and used to measure alanine and aspartate aminotransferase (ALT and AST) levels for evaluating liver function. For histopathological analysis, upon reaching the tumor size endpoint, major organs were harvested from mice in each treatment group, fixed in 10% neutral buffered formalin (in PBS), embedded in paraffin, and sectioned into 5 µm slices for hematoxylin and eosin (H&E) staining. Body weight change (%) was calculated as [(W_1_ ‐ W_0_) / W_0_] × 100%. All samples were sent to Wuhan Servicebio Technology Co., Ltd. for analysis and data collection.

For the assessment of the biodistribution and metabolism of MnSTF in vivo, male C57BL/6 mice received subcutaneous injections of 100 µL of either 5 mM MnCl_2_ or MnSTF every other day, totaling three administrations. Fourteen days post the final injection, major organs (heart, liver, spleen, lung, kidney, and brain) were collected and weighed. The tissues were digested in aqua regia at room temperature until a clear solution formed, and the manganese content was subsequently quantified by inductively coupled plasma mass spectrometry (ICP‐MS).

### Statistical Analysis

GraphPad Prism version 9.5.1 (GraphPad Software, San Diego, CA, USA) was used for all statistical analyses. All data are presented as mean ± standard deviation (SD). Statistical differences between two independent groups were analyzed using a two‐tailed unpaired t‐test. For comparisons among multiple groups, one‐way or two‐way analysis of variance (ANOVA) was used. Statistical significance was indicated as follows: *p < 0.05, **p < 0.01, ***p < 0.001, ****p < 0.0001; ns, not significant.

## Conflict of Interest

The authors declare no conflict of interest.

## Author Contributions

G.S. and J.P. contributed equally to this work. G.F.S., J.C.P., and A.H.Y. conceived and designed the experiments. G.F.S., J.C.P., R.L., and R.X.L. performed the experiments and analyzed the data. Z.Y.L. prepared the figures. G.F.S. and A.H.Y. wrote the manuscript. Y.Q.H., A.H.Y., and T.S.L. supervised the project. All authors discussed the results and approved the final manuscript.

## Supporting information



Supporting Information

## Data Availability

The data that support the findings of this study are available in the supplementary material of this article.
