# Supplementary material for: Soft‐Drug‐Inspired MnSTF Nano‐Adjuvant for Safe and Synergistic cGAS–STING Activation in Tumor Immunotherapy
Source: Adv Sci (Weinh). 2025 Dec 16;13(9):e15432. doi: 10.1002/advs.202515432 (PMC12904058; doi:10.1002/advs.202515432)
Supplement: Supplementary file 1 — Supporting Information [file ADVS-13-e15432-s001.docx]

Supporting Information

**Soft-Drug-Inspired MnSTF Nano-Adjuvant for Safe and Synergistic cGAS–STING Activation in Tumor Immunotherapy**

*Guangfei Sun^1†^, Jiancheng Pan^1†^, Rui Li^1^, Ruoxi Li^1^, Ziyan Liu^1^, Jinhui Wu^1,3^, Tingsheng Lin^2*^, Yiqiao Hu^1,3*^, Ahu Yuan^1,3*^*

^1^ State Key Laboratory of Pharmaceutical Biotechnology, Medical School and School of Life Science, Nanjing University, Nanjing 210093, China.

^2^ Department of Urology, Nanjing Drum Tower Hospital, The Aﬃliated Hospital of Nanjing University Medical School, Nanjing 210093, P. R. China

^3^ Jiangsu Key Laboratory for Nano Technology, Nanjing University, Nanjing 210093, China.

^†^These authors contributed equally to this work.

^*^Corresponding author's E-mail: dr_lts@nju.edu.cn (T.L.); [huyiqiao@nju.edu.cn](mailto:huyiqiao@nju.edu.cn) (Y.H.) and yuannju@nju.edu.cn (A.Y.).

**Chemicals and Solvents**

All chemicals involved in the synthesis of the target compounds were of reagent grade and used as received without further purification, unless otherwise specified. 1-Boc-4-(2-hydroxyethyl)piperidine (Bidepharm, Shanghai, China), dry dichloromethane (DCM; Adamas, Shanghai, China), iodine (Adamas, Shanghai, China), triphenylphosphine (Adamas, Shanghai, China), imidazole (Adamas, Shanghai, China), sodium thiosulfate (Adamas, Shanghai, China), diethyl phosphite (Adamas, Shanghai, China), cesium carbonate (Cs_2_CO_3_; Bidepharm, Shanghai, China), dry N,N-dimethylformamide (DMF; Adamas, Shanghai, China), ethyl acetate (Bidepharm, Shanghai, China), trifluoroacetic acid (TFA; Adamas, Shanghai, China), N,N-diisopropylethylamine (DIPEA; Adamas, Shanghai, China), 4-chloro-8-methoxyquinoline (Bidepharm, Shanghai, China), bromotrimethylsilane (Adamas, Shanghai, China), acetonitrile (CH_3_CN; Adamas, Shanghai, China), tetrahydrofuran (THF; Adamas, Shanghai, China), 5,6-dimethoxybenzo[b]thiophene-2-carboxylic acid (Adamas, Shanghai, China), oxalyl chloride (Adamas, Shanghai, China), copper(I) thiophene-2-carboxylate (TCI, Tokyo, Japan), (3-ethoxy-3-oxopropyl)zinc(II) bromide (Sigma-Aldrich, Shanghai, China), lithium hydroxide (Adamas, Shanghai, China), manganese(II) chloride tetrahydrate (Adamas, Shanghai, China), indocyanine green (ICG; MedChemExpress, Monmouth Junction, NJ, USA), and ovalbumin (OVA; Meryer, Shanghai, China), were used in the synthesis. The antigenic peptides (SIINFEKL, SVYDFFVWL, SIYRYYGL, and KVPRNQDWL) were purchased from GenScript.

**Synthesis of STF-1623.**

Preparation of **S1**^[1]^


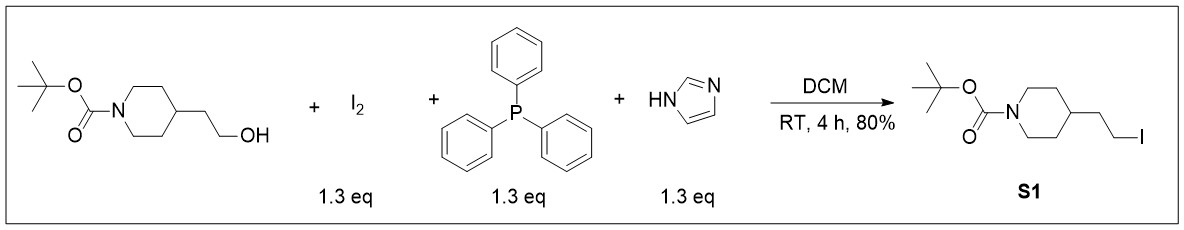


Iodine (3.96 g, 15.6 mmol), triphenylphosphine (4.09 g, 15.6 mmol), and imidazole (1.06 g, 15.6 mmol) were suspended in 60 mL of dry dichloromethane (DCM) and stirred at room temperature in the dark for 30 min. 1-Boc-4-(2-hydroxyethyl)piperidine (2.75 g, 12.0 mmol) was dissolved in 15 mL of dry DCM and added dropwise to the reaction mixture. The mixture was stirred for an additional 4 h. After completion, the reaction mixture was extracted with DCM, washed successively with saturated aqueous sodium thiosulfate solution, water, and brine, dried over anhydrous Na₂SO₄, and concentrated under reduced pressure. The crude product was purified by flash chromatography to afford 1-piperidinecarboxylic acid, 4-(2-iodoethyl)-, 1,1-dimethylethyl ester (**S1**) in 80% yield.

Preparation of **S2**^[2]^


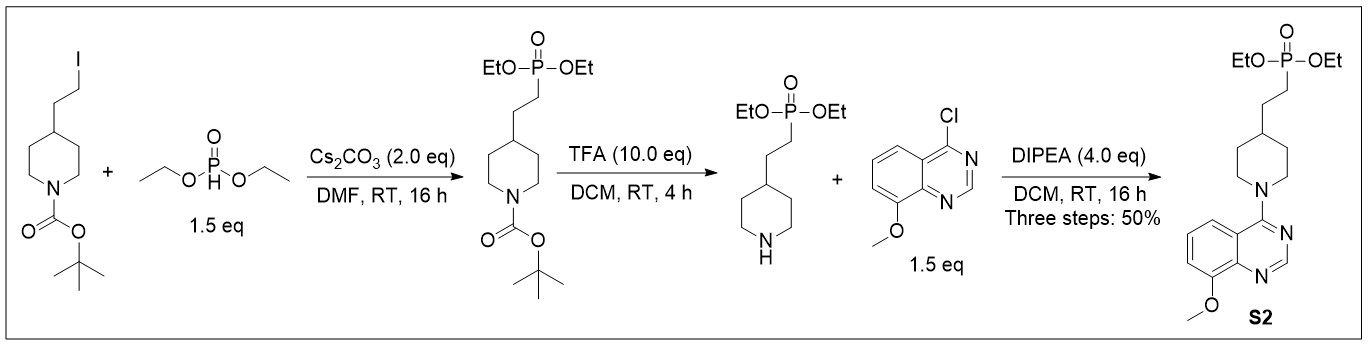


1-Piperidinecarboxylic acid, 4-(2-iodoethyl)-, 1,1-dimethylethyl ester (**S1**, 2.0 g, 5.9 mmol), diethyl phosphite (1.1 mL, 8.8 mmol), and cesium carbonate (Cs_2_CO_3_, 3.8 g, 11.8 mmol) were suspended in DMF (15 mL) and stirred at room temperature under a dry nitrogen atmosphere for 16 h. The reaction mixture was extracted with ethyl acetate, washed with water and brine, dried over anhydrous Na_2_SO_4_, and concentrated under reduced pressure. The resulting crude product was used directly in the next step without further purification.

The crude product was dissolved in DCM (25 mL), and TFA (4.0 mL, 59 mmol) was added dropwise over 10 min. The reaction mixture was stirred at room temperature for 4 h. The solvent was removed under reduced pressure to afford Diethyl (2-(piperidin-4-yl)ethyl)phosphonate as a crude product, which was used directly in the next step without purification.

A mixture of the crude product and 4-chloro-8-methoxyquinoline (1.7 g, 8.8 mmol) was dissolved in dry DCM (25 mL), and DIPEA (4.1 mL, 23.6 mmol) was added dropwise. After stirring at room temperature for 16 h, the reaction was quenched by the addition of saturated aqueous ammonium chloride solution. The mixture was extracted with DCM, washed with water and brine, dried over anhydrous Na_2_SO_4_, and concentrated under reduced pressure. The crude product was purified by silica gel column chromatography to afford **S2** (50% yield over three steps).

Preparation of P-[2-[1-(8-methoxyquinazolin-4-yl)piperidin-4-yl]ethyl]phosphonic acid (**STF-1623**) ^[3]^


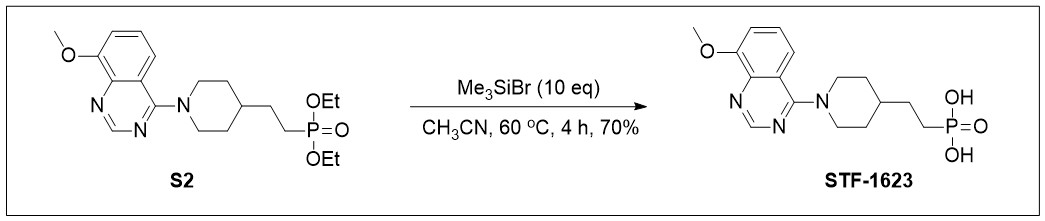


The compound **S2** (1.0 g, 2.45 mmol) was dissolved in 25 mL of CH_3_CN, and bromotrimethylsilane (3.2 mL, 24.5 mmol) was added dropwise. The reaction mixture was stirred at 60 °C for 4 h and then cooled to room temperature. The reaction was quenched by the addition of 10 mL MeOH, followed by stirring for 1 h. The solvent was removed under reduced pressure, and the residue was purified by silica gel column chromatography to afford the final product **STF-1623** in 70% yield. HRMS [ESI] m/z Calcd for C_16_H_22_N_3_O_4_P [M-H]^-^ Exact Mass: 350.13; Found 350.1265. ^1^H NMR (600 MHz, Methanol-*d_4_*) δ 8.54 (s, 1H), 7.71 (d, *J* = 8.4 Hz, 1H), 7.67 (t, *J* = 8.4 Hz, 1H), 7.57 (d, *J* = 8.4 Hz, 1H), 4.12 (s, 3H) , 2.05 (d, *J* = 12.0 Hz, 2H), 1.93-1.87 (m, 1H), 1.81 – 1.75 (m, 2H), 1.67 – 1.61 (m, 2H), 1.48 – 1.41 (m, 2H). ^13^C NMR (150 MHz, DMSO-*d_6_*) δ 161.86, 148.90, 148.56, 131.23, 127.69, 118.62, 115.55, 112.78, 57.32, 40.51, 35.69, 35.58, 31.96, 29.42 (d, ^1^*J*_C-P_ = 4.2 Hz), 25.72, 24.81. The ^1^H and ^13^C NMR spectra were recorded on a Bruker DRX-600 spectrometer (600 MHz, 150 MHz). High-resolution mass spectrometry (HRMS) data were acquired using an AB SCIEX TripleTOF 4600 mass spectrometer (**Supplementary Figs. 13, 14**).

**Synthesis of MSA-2**^[4]^

Preparation of 5,6-dimethoxybenzo[b]thiophene-2-carbonyl chloride


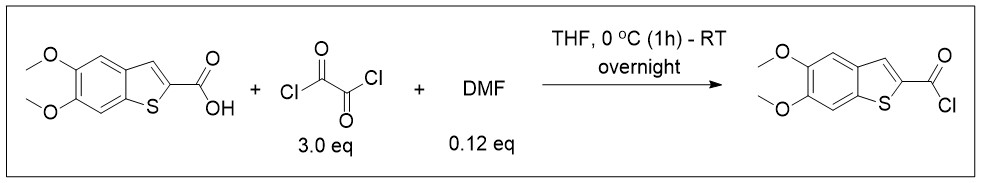


5,6-Dimethoxybenzo[b]thiophene-2-carboxylic acid (0.4 g, 1.68 mmol) was dissolved in THF (16 mL) and cooled in an ice bath under an argon atmosphere. Then, oxalyl chloride (0.45 mL, 5.04 mmol) and DMF (16 µL, 0.2 mmol) were added sequentially. The reaction mixture was stirred at 0 °C for 1 h, then warmed to room temperature and stirred overnight. The mixture was concentrated to dryness under reduced pressure, and the resulting 5,6-dimethoxybenzo[b]thiophene-2-carbonyl chloride was used directly in the next step without purification.

Preparation of Ethyl 4-(5,6-dimethoxybenzo[b]thiophen-2-yl)-4-oxobutanoate


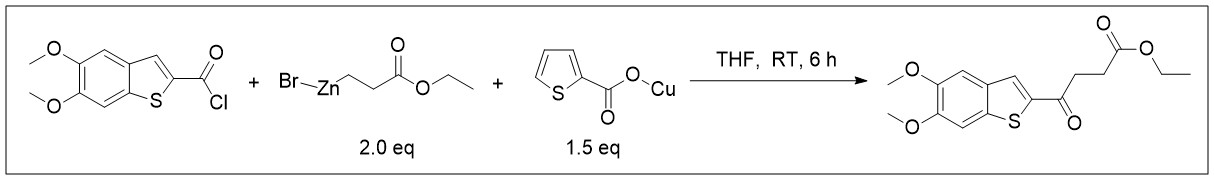


Copper(I) thiophene-2-carboxylate (480 mg, 2.52 mmol) was suspended in dry THF (2 mL), and (3-ethoxy-3-oxopropyl)zinc(II) bromide (0.5 M in THF, 3.4 mL, 3.36 mmol) was added. The reaction mixture was stirred at 0 °C for 20 min, followed by the dropwise addition of 5,6-dimethoxybenzo[b]thiophene-2-carbonyl chloride (1.68 mmol) in THF (3.5 mL). The resulting mixture was allowed to warm to room temperature and stirred for 6 h. Then, 2 mL of saturated aqueous ammonium chloride was added to quench the reaction. The mixture was extracted with ethyl acetate, washed with water and brine, dried over anhydrous Na₂SO₄, and concentrated under reduced pressure. The residue was purified by silica gel column chromatography to afford ethyl 4-(5,6-dimethoxybenzo[b]thiophen-2-yl)-4-oxobutanoate in 51% yield over two steps. ^1^H NMR (600 MHz, CDCl_3_) δ 7.88 (s, 1H), 7.24 (s, 1H), 7.24 (s, 1H), 4.16 (q, *J* = 7.2 Hz, 2H), 3.97 (s, 3H), 3.95 (s, 3H), 3.31 (t, *J* = 7.2 Hz, 2H), 2.77 (t, *J* = 6.6 Hz, 2H), 1.26 (t, *J* = 7.2 Hz, 3H) (**Supplementary Fig. 15a**). The ^1^H NMR data are consistent with those reported in the literature.

Preparation of 4-(5,6-dimethoxybenzo[b]thiophen 2-yl)-4-oxobutanoic acid (**MSA-2**)


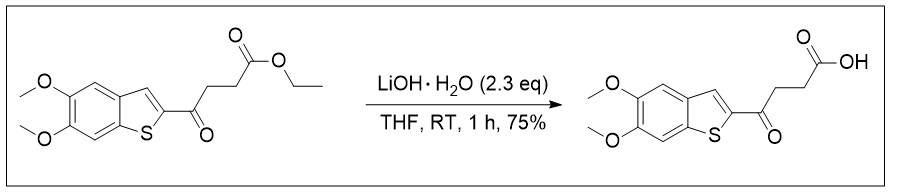


Ethyl 4-(5,6-dimethoxybenzo[b]thiophen-2-yl)-4-oxobutanoate (138 mg, 0.248 mmol) was suspended in dry THF (1 mL), and lithium hydroxide (1.0 M in water, 1 mL, 1.00 mmol) was added. The reaction mixture was stirred at room temperature for 1 h, then HCl (1.0 M in water, 1 mL) was added. A precipitate formed within 30 min and was collected by centrifugation to afford 4-(5,6-dimethoxybenzo[b]thiophen-2-yl)-4-oxobutanoic acid (95 mg, 75% yield). ^1^H NMR (600 MHz, DMSO-*d_6_*) δ 12.20 (br s, 1H), 8.20 (s, 1H), 7.60 (s, 1H), 7.48 (s, 1H), 3.86 (s, 3H), 3.84 (s, 3H), 3.26 (t, *J* = 6.0 Hz, 2H), 2.60 (t, *J* = 6.0 Hz, 2H) (**Supplementary Fig. 15b**). The ^1^H NMR data are consistent with those reported in the literature.


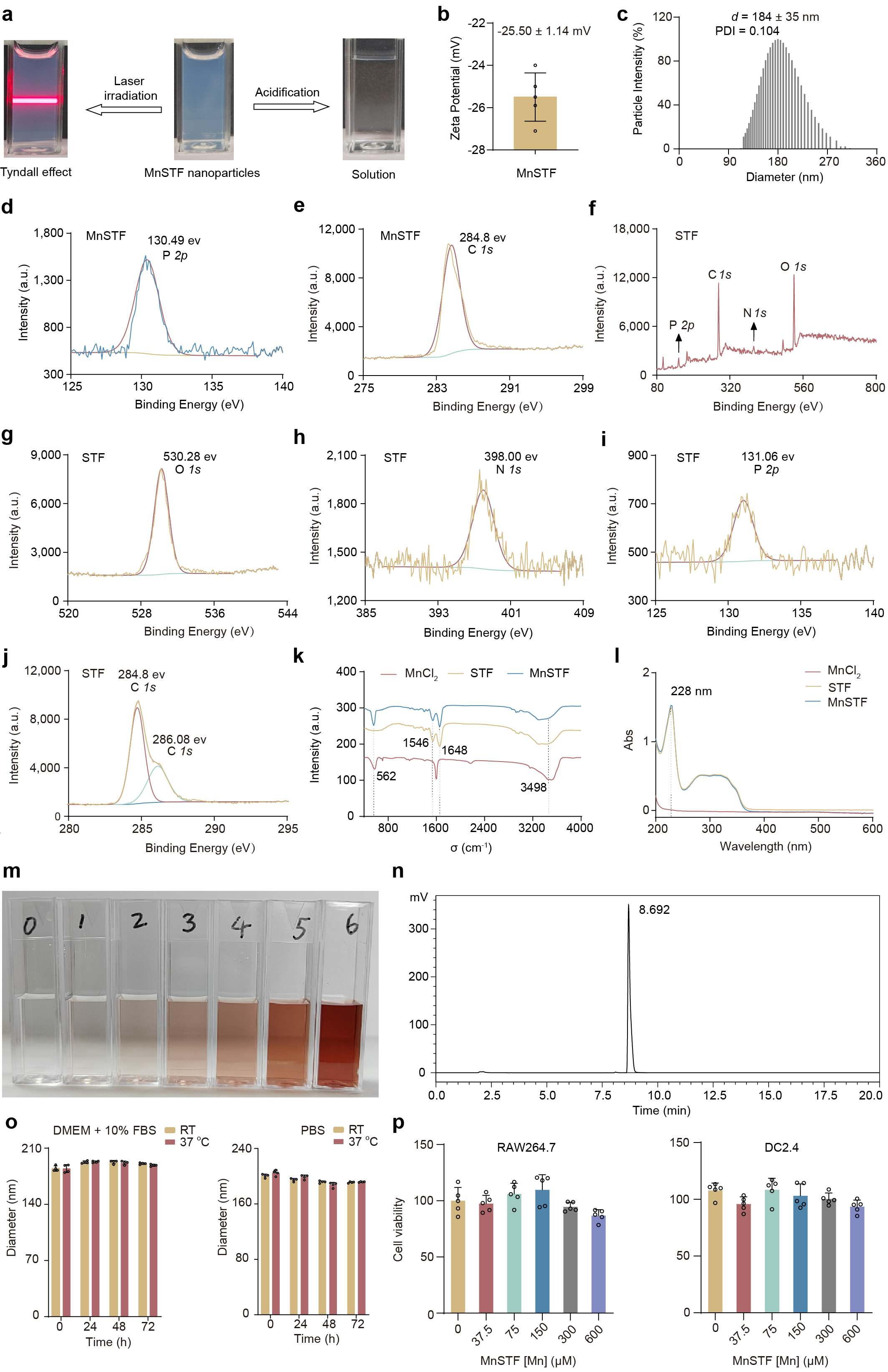


**Supplementary Fig. 1 |** **Characterizations and properties of MnSTF.** (**a**) Tyndall effect and acidification of MnSTF aqueous suspension. (**b**) Zeta potential of MnSTF aqueous suspension (n = 5). (**c**) Size distribution of MnSTF as measured by dynamic light scattering (DLS). PDI, polydispersity index; d, diameter. High-resolution XPS spectra of P *2p* (**d**) and C *1s* (**e**) of MnSTF. (**f**) Full X-ray photoelectron spectroscopy (XPS) spectrum of STF. High resolution O *1s* (**g**), N *1s* (**h**), P *2p* (**i**) and C *1s* (**j**) XPS spectra of STF. (**k**) Fourier transform infrared (FT-IR) spectra of MnCl_2_, STF and MnSTF. (**l**) The ultraviolet-visible (UV-vis) spectra of MnCl_2_, STF and MnSTF in deionized water. (**m**) Representative image of the reaction between Mn^2+^ and formaldehyde oxime at different concentrations. (**n**) Representative HPLC chromatogram of STF. (**o**) Stability of MnSTF in 10% FBS and PBS at room temperature and 37 °C. (**p**) Cell viability of RAW264.7 and DC2.4 cells after treatment with MnSTF.


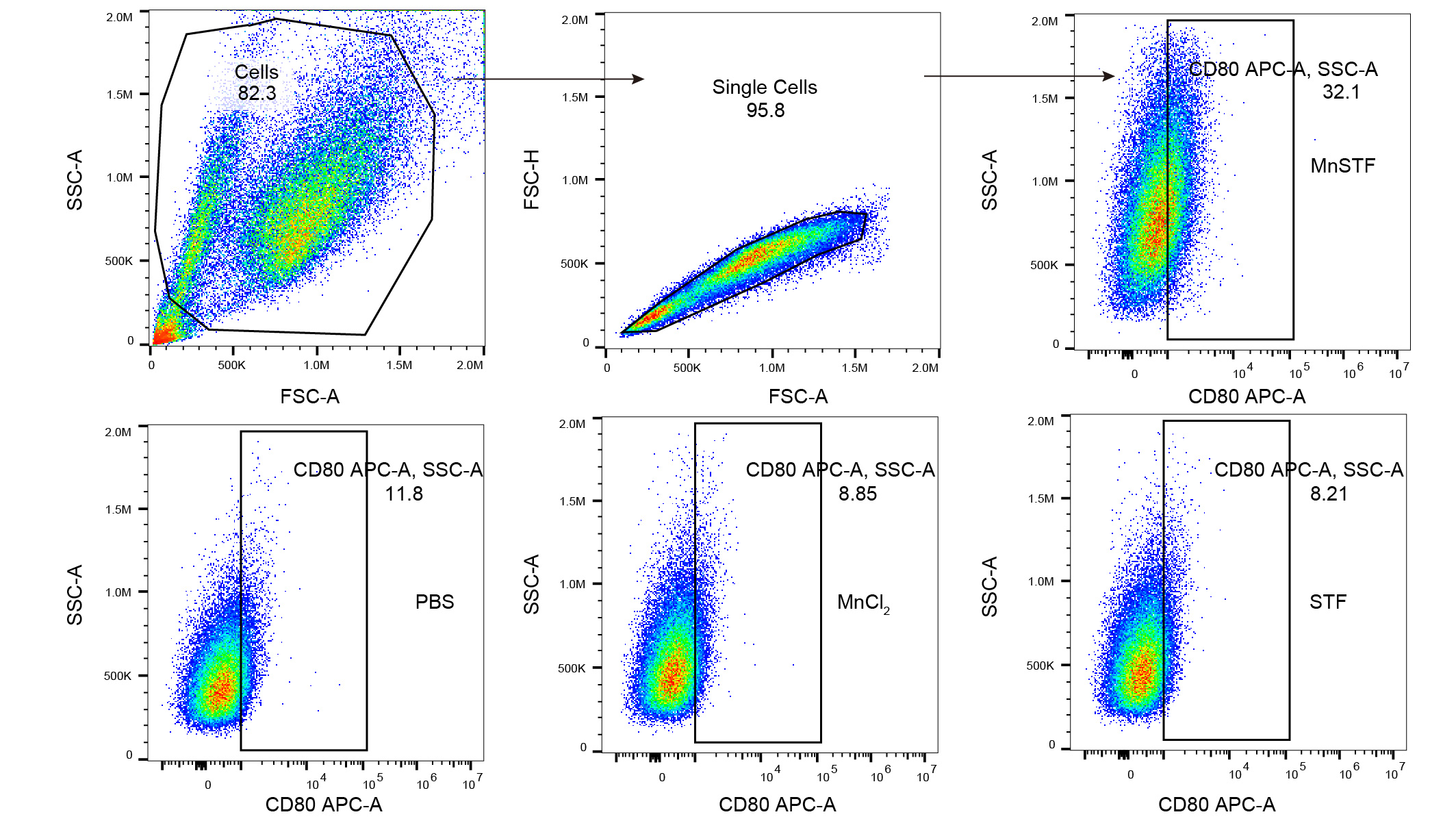


**Supplementary Fig. 2 |** Representative Gating strategy for CD80^+^ DC2.4 analysis as seen in **Fig. 3h**.


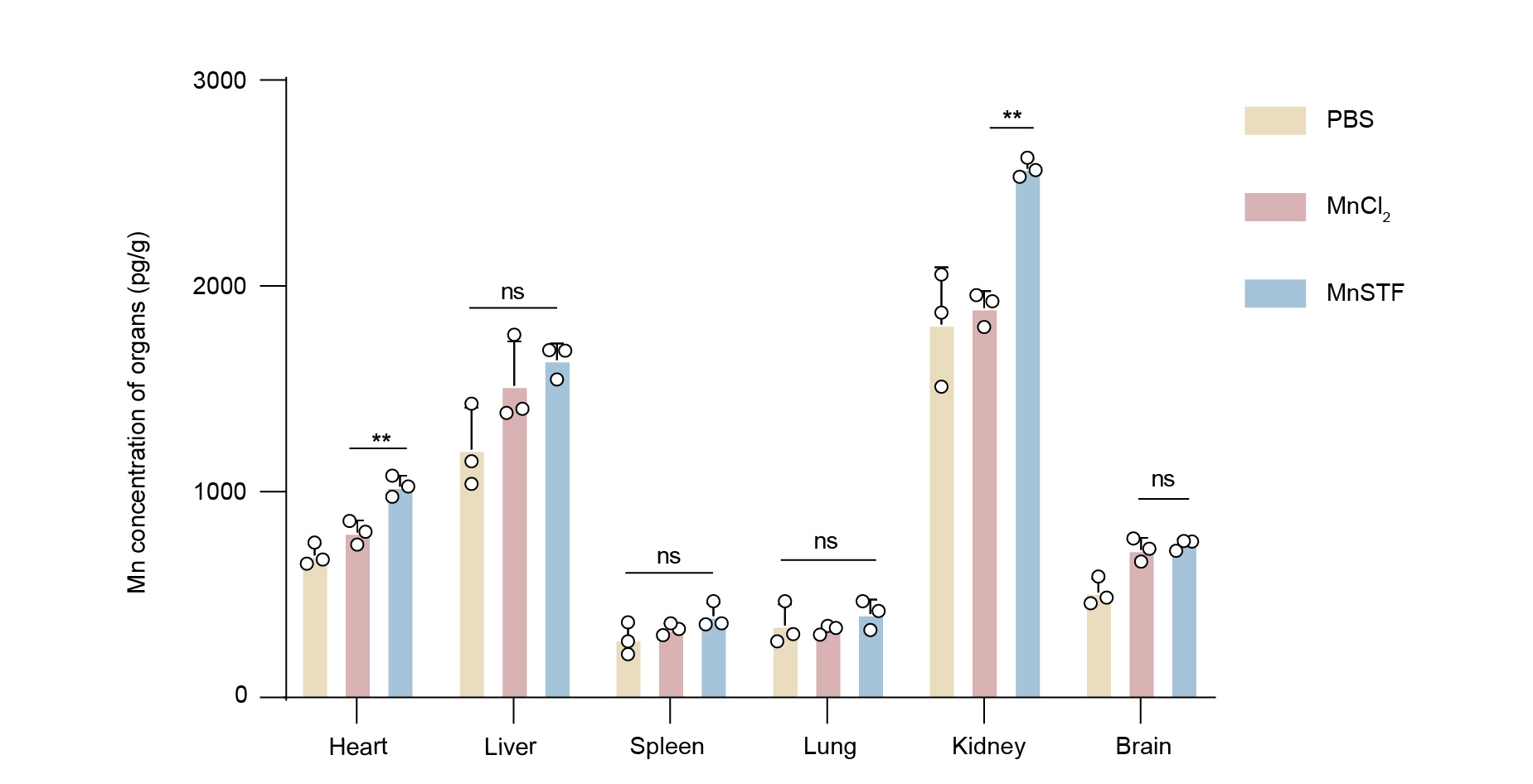


**Supplementary Fig. 3 | Manganese biodistribution.**Mice received three doses of MnSTF or MnCl_2_ every other day. Mn concentrations in organs were measured by ICP-MS 14 days after the final dose.


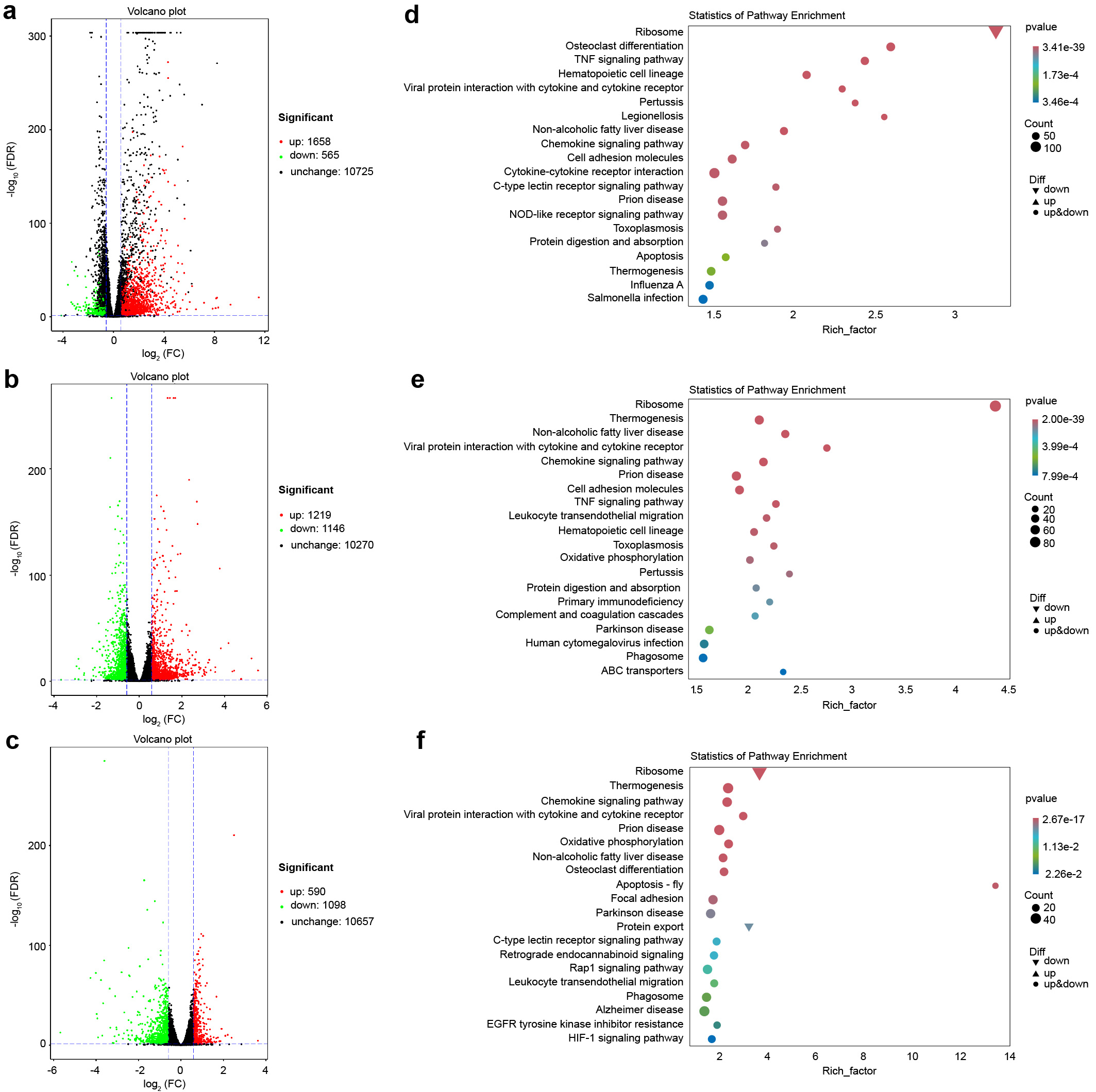


**Supplementary Fig. 4 |** **RNA sequencing of B16F10-OVA tumor tissues treated with MnSTF.** Volcano plot analysis of differential gene expression in the tumor on days 1 (**a**), 3 (**b**), and 5 (**c**) after treatment. GO enrichment analysis in the tumor on days 1 (**d**), 3 (**e**), and 5 (**f**) after treatment.


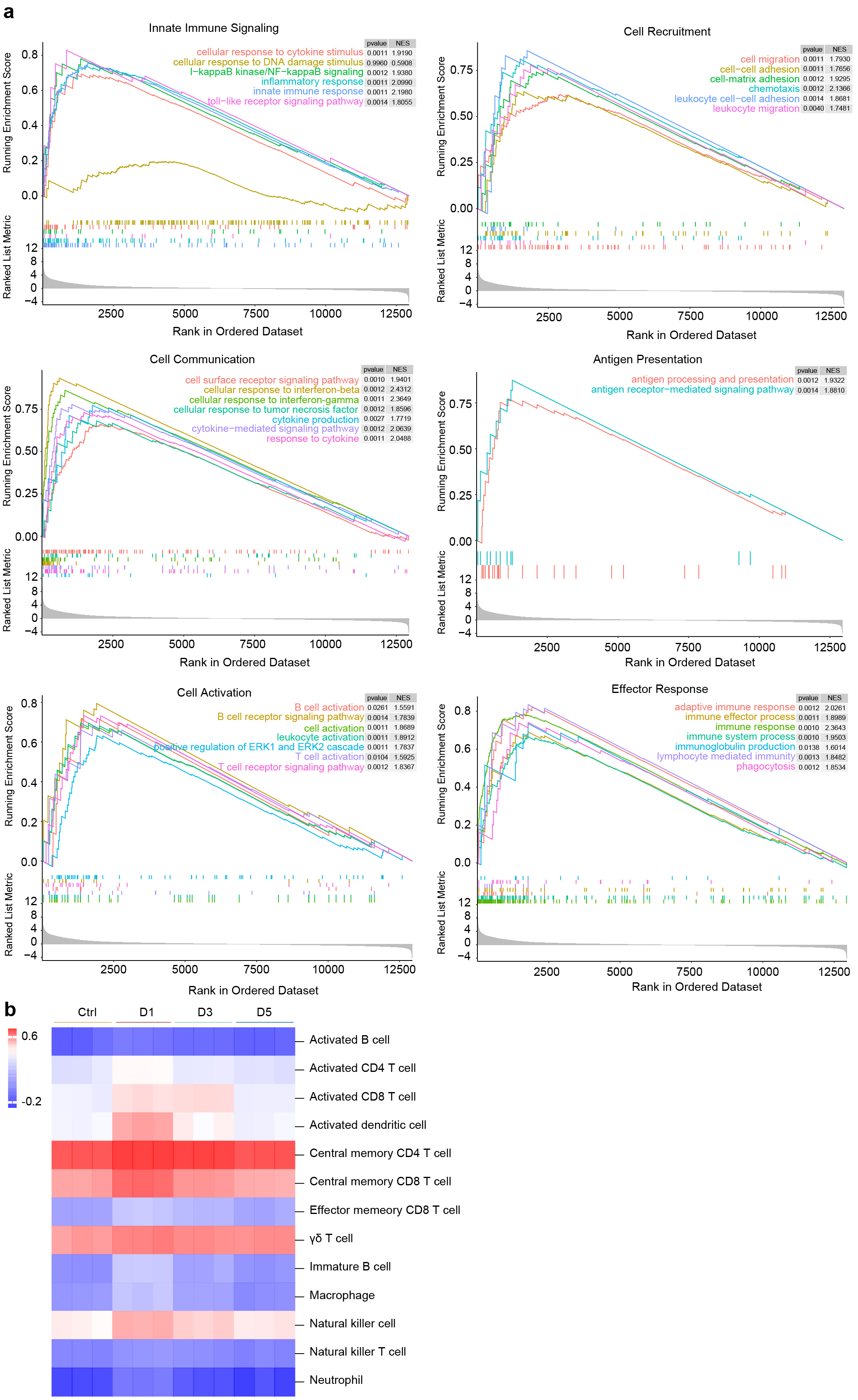


**Supplementary Fig. 5 |** **RNA sequencing of B16F10-OVA tumor tissues treated with MnSTF.** (**a**) GSEA of tumors from the treatment group versus the PBS control at day 1 post-treatment. (**b**) Heatmap of immune cell deconvolution in tumors at days 1 (D1), 3 (D3), and 5 (D5) post-treatment.


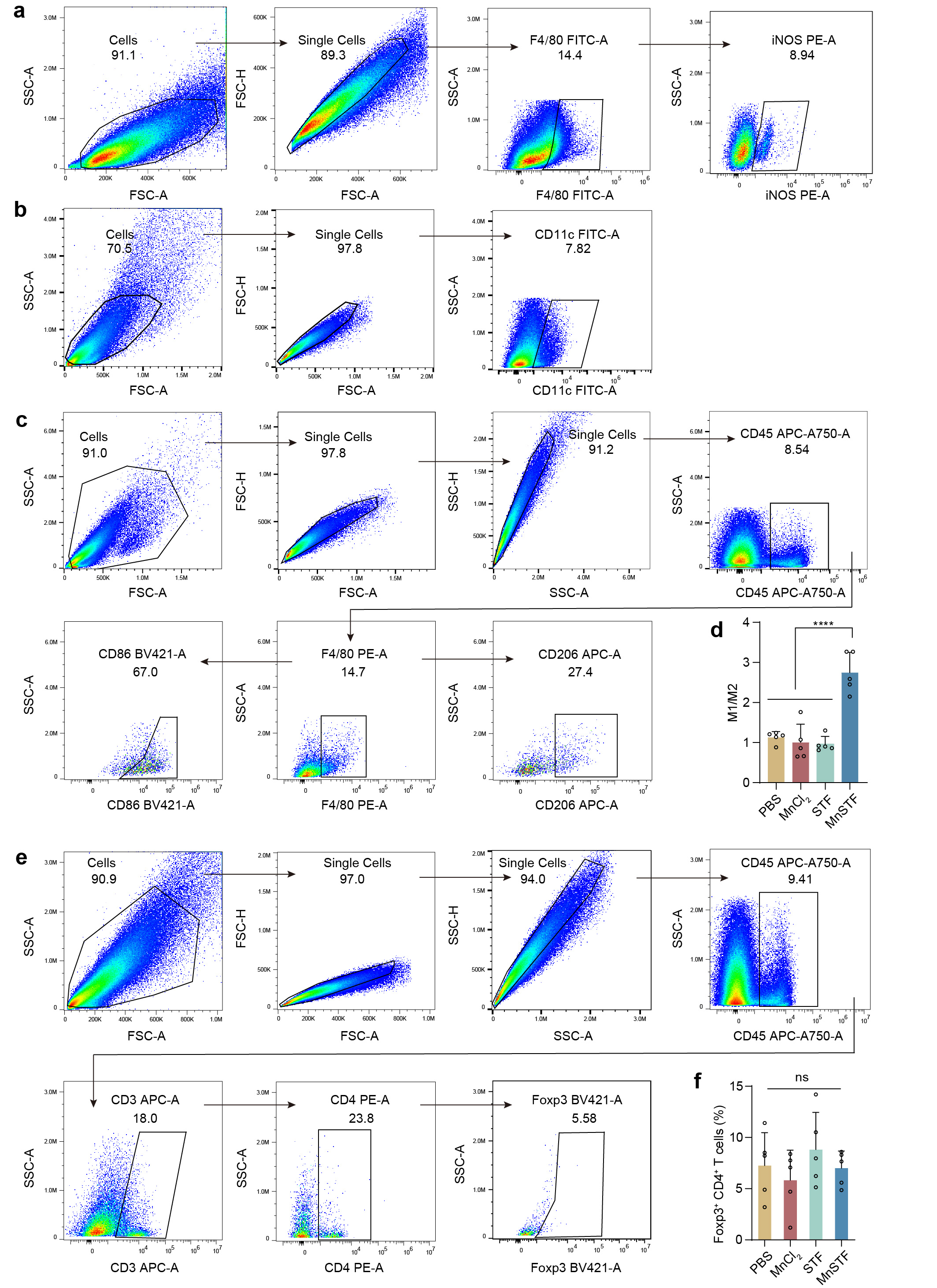


**Supplementary Fig. 6 |** (**a**) Gating strategy of iNOS^+^ F4/80^+^ cells in the tumor as seen in **Fig. 5f**. (**b**) Gating strategy of CD11^+^ cells in the tumor as seen in **Fig. 5h**. (**c**) Gating strategy for the identification of M1-like (F4/80⁺CD86⁺) and M2-like (F4/80⁺CD206⁺) macrophages in tumor tissues. (**d**) Quantification of the M1/M2 ratio (n = 5). (**e**) Gating strategy of the Treg (Foxp3^+^CD4^+^) in tumor tissues. (**f**) Quantification of the Treg proportion (n = 5).


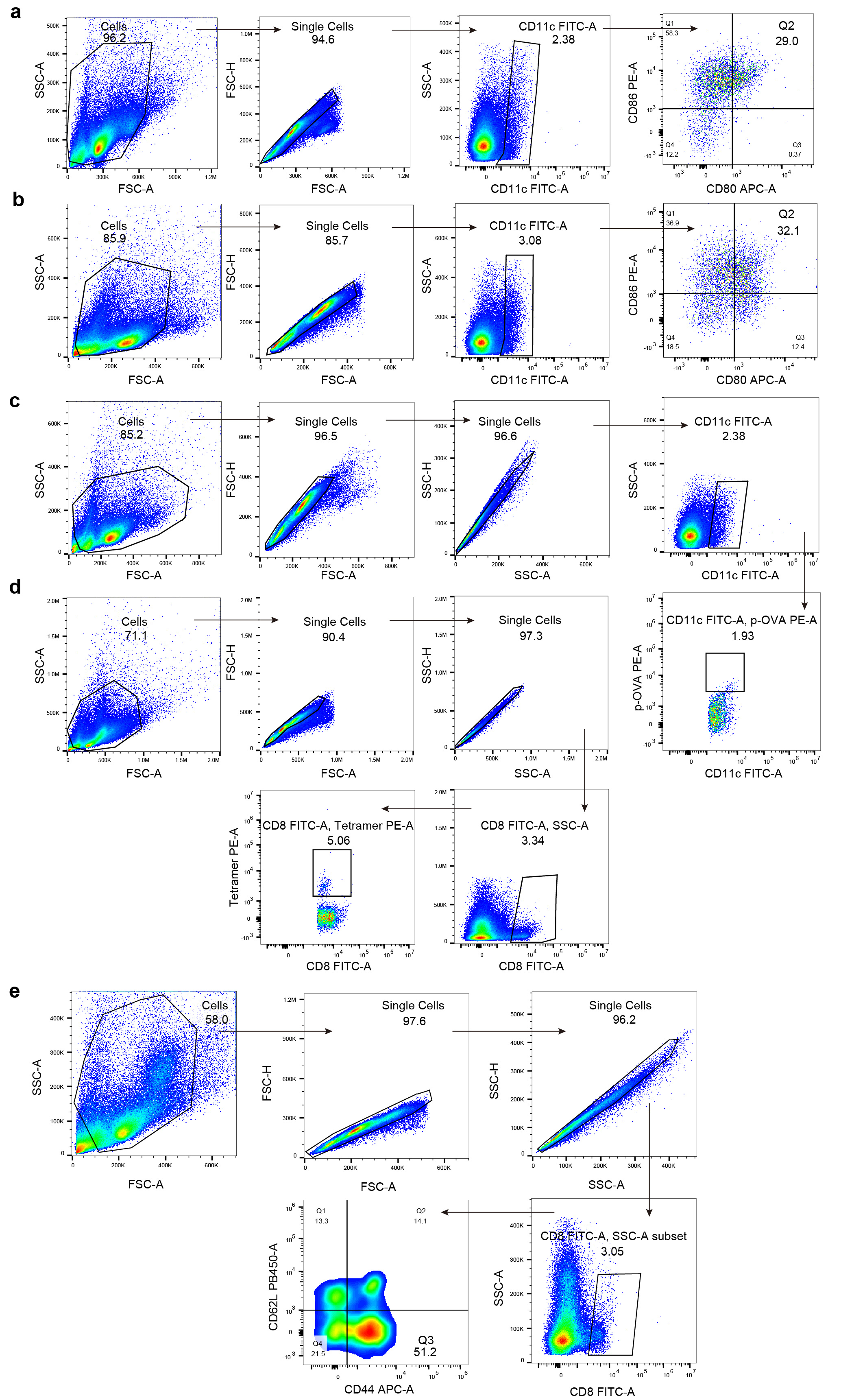


**Supplementary Fig. 7 |** (**a, b**) Gating strategy of CD80^+^ CD86^+^ in CD11c^+^ cells in lymph node as seen in **Fig. 5j** (**a**) and **Fig. 6g** (**b**). (**c**) Gating strategy of SIINFEKL–H-2K^b+^ cells in CD11c^+^ DCs in lymph node as seen in **Fig. 6e**. (**d**) Gating strategy of SIINFEKL tetramer^+^ CD8^+^ T cells among spleens of mice in **Fig. 7d**. (**e**) Gating strategy for effector memory T cells (TEM cells) and central memory T cells (TCM cells) analysis as seen in **Fig. 8g.**


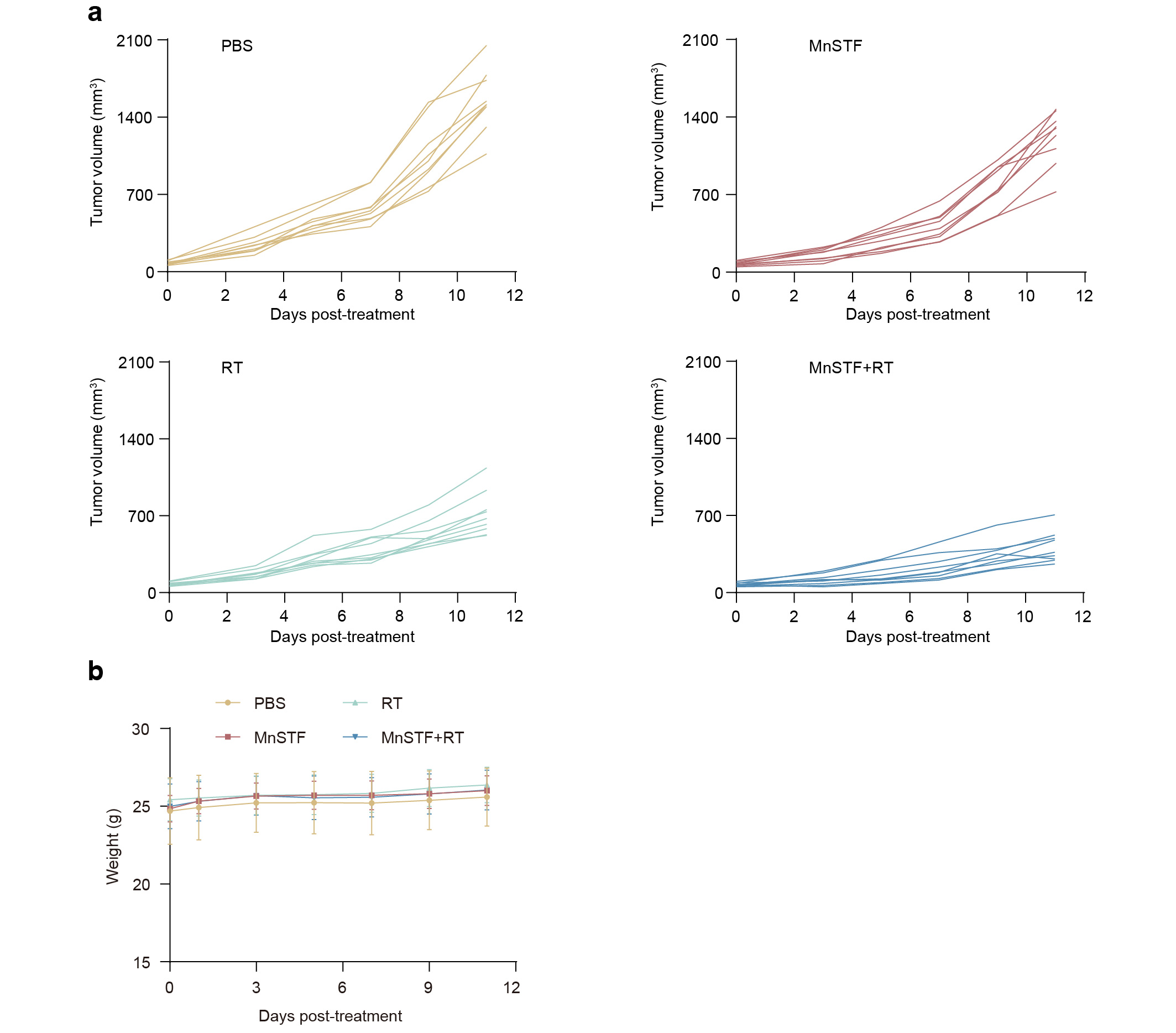


**Supplementary Fig. 8 |** (**a**) Growth curves of individual B16F10-OVA tumors in the PBS, MnSTF, RT or MnSTF + RT groups (n = 9). (**b**) Body weight changes of mice in the PBS, MnSTF, RT, and MnSTF + RT groups (n = 9).


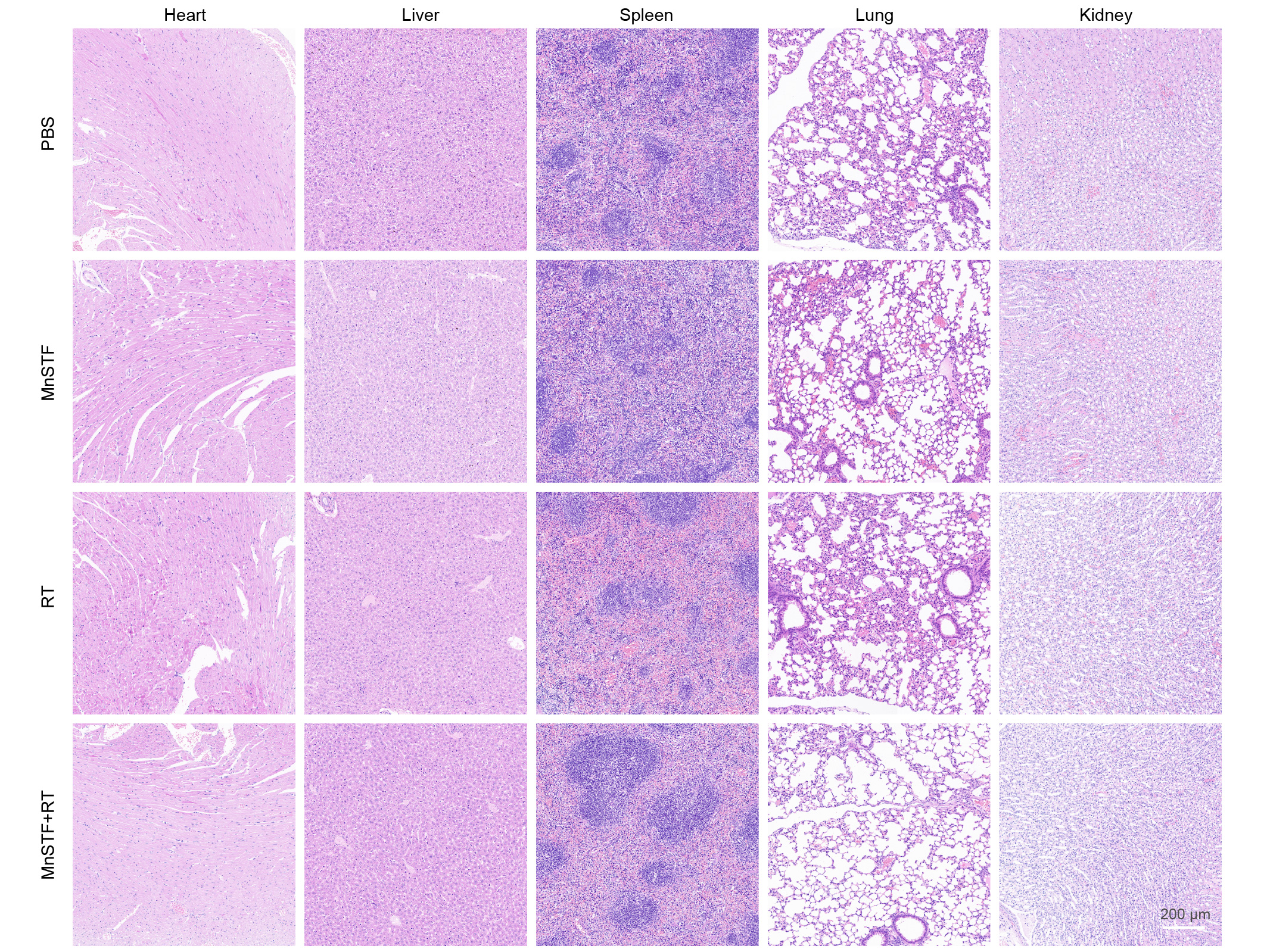


**Supplementary Fig. 9 |** Histological examination (H&E) staining of heart, liver, spleen, lung and kidney sections of the PBS, MnSTF, RT, or MnSTF + RT groups (n = 3), scale bar = 200 μm.


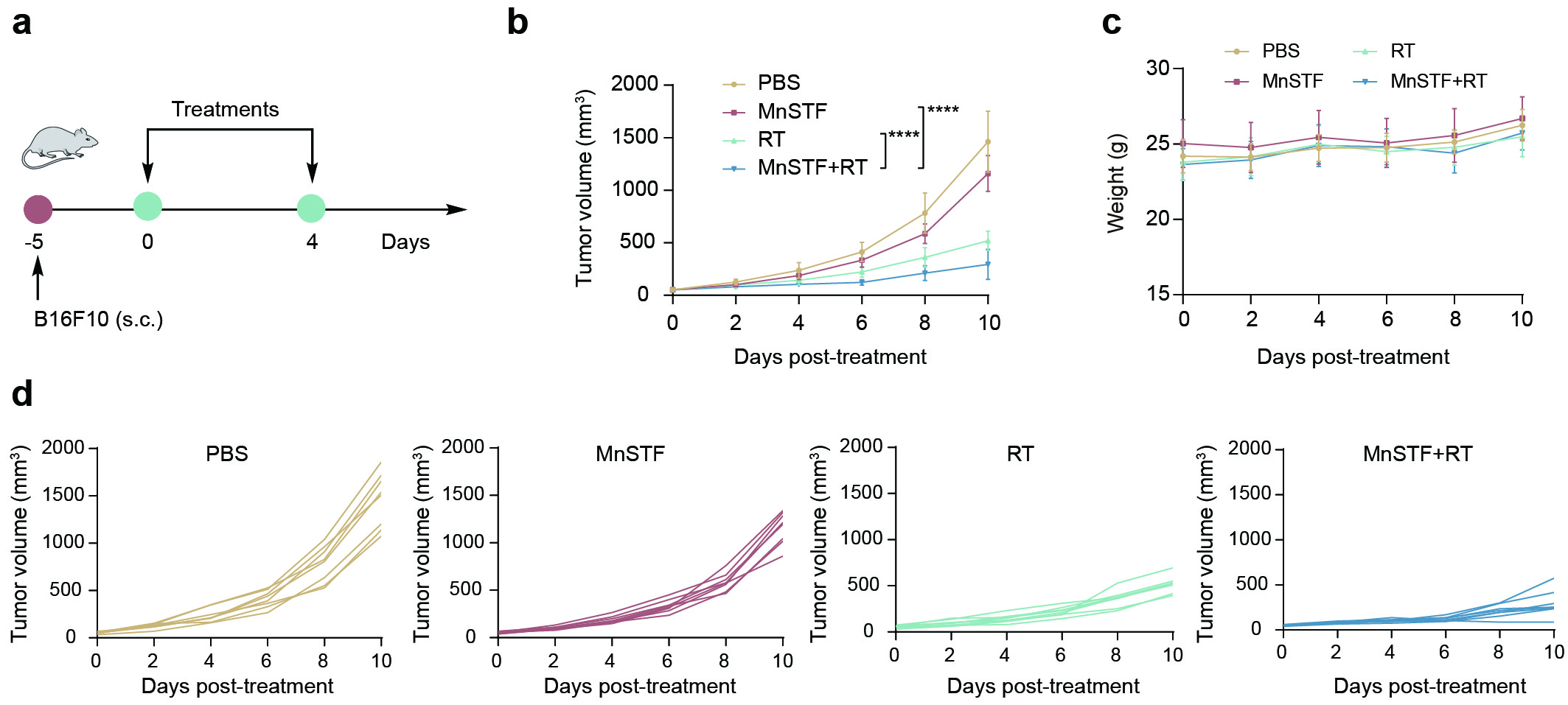


**Supplementary Fig. 10 |** (**a**) Experimental timeline for B16F10 tumor-bearing mice treated with PBS, RT (6 Gy), MnSTF (0.69 µmol Mn equivalent), or their combination (RT + MnSTF). Treatments were administered every four days for two doses. (**b**) Tumor growth kinetics following different treatments (n = 8). (**c**) Body weight changes of mice in the PBS, MnSTF, RT, or MnSTF +RT groups (n = 8). (**d**) Growth curves of individual B16F10 tumors in the PBS, MnSTF, RT or MnSTF + RT groups (n = 8).


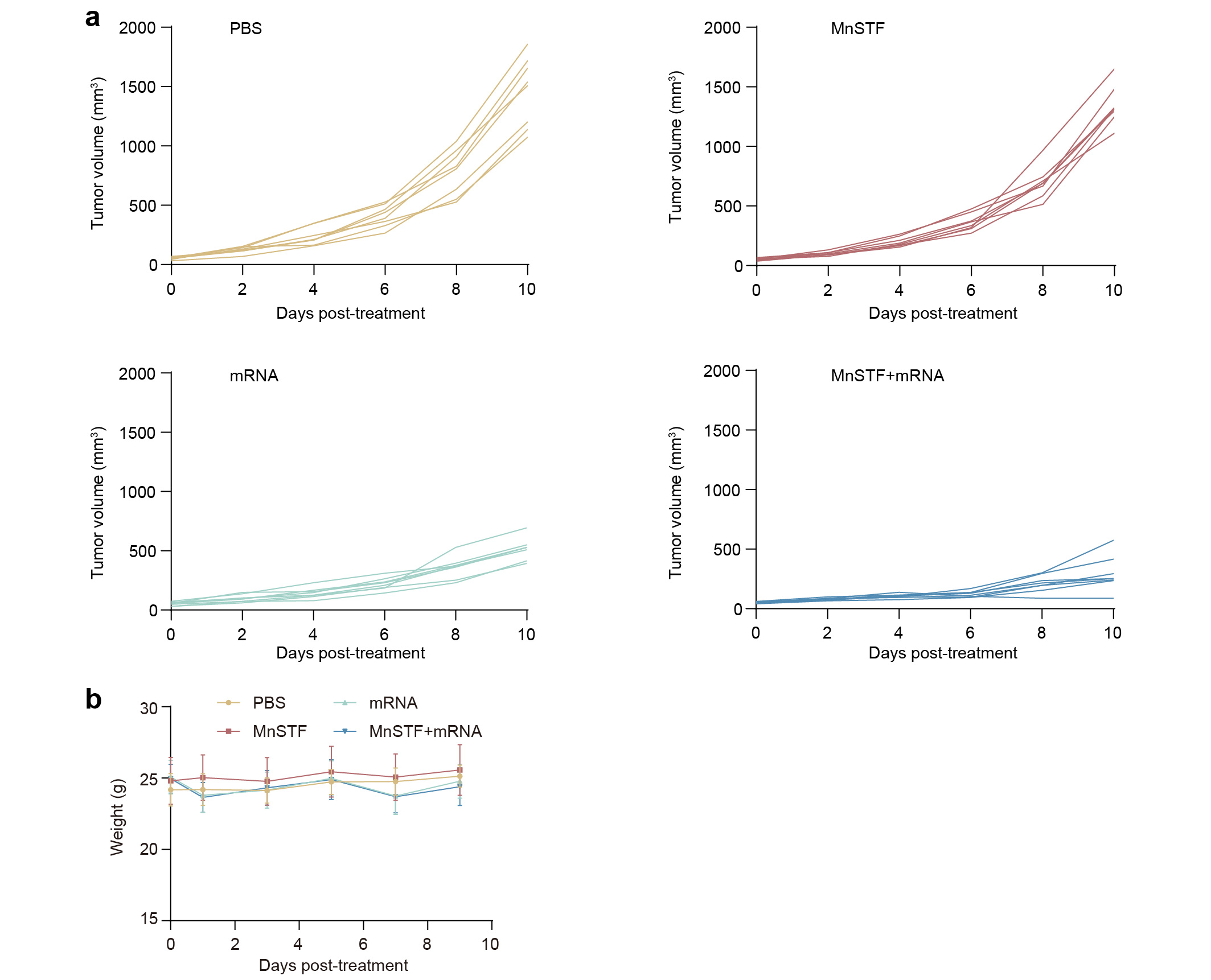


**Supplementary Fig. 11 |** (**a**) Growth curves of individual B16F10-OVA tumors in the PBS, MnSTF, mRNA or MnSTF + mRNA groups (n = 8). (**b**) Body weight changes of mice in the PBS, MnSTF, mRNA, or MnSTF + mRNA groups (n = 8).


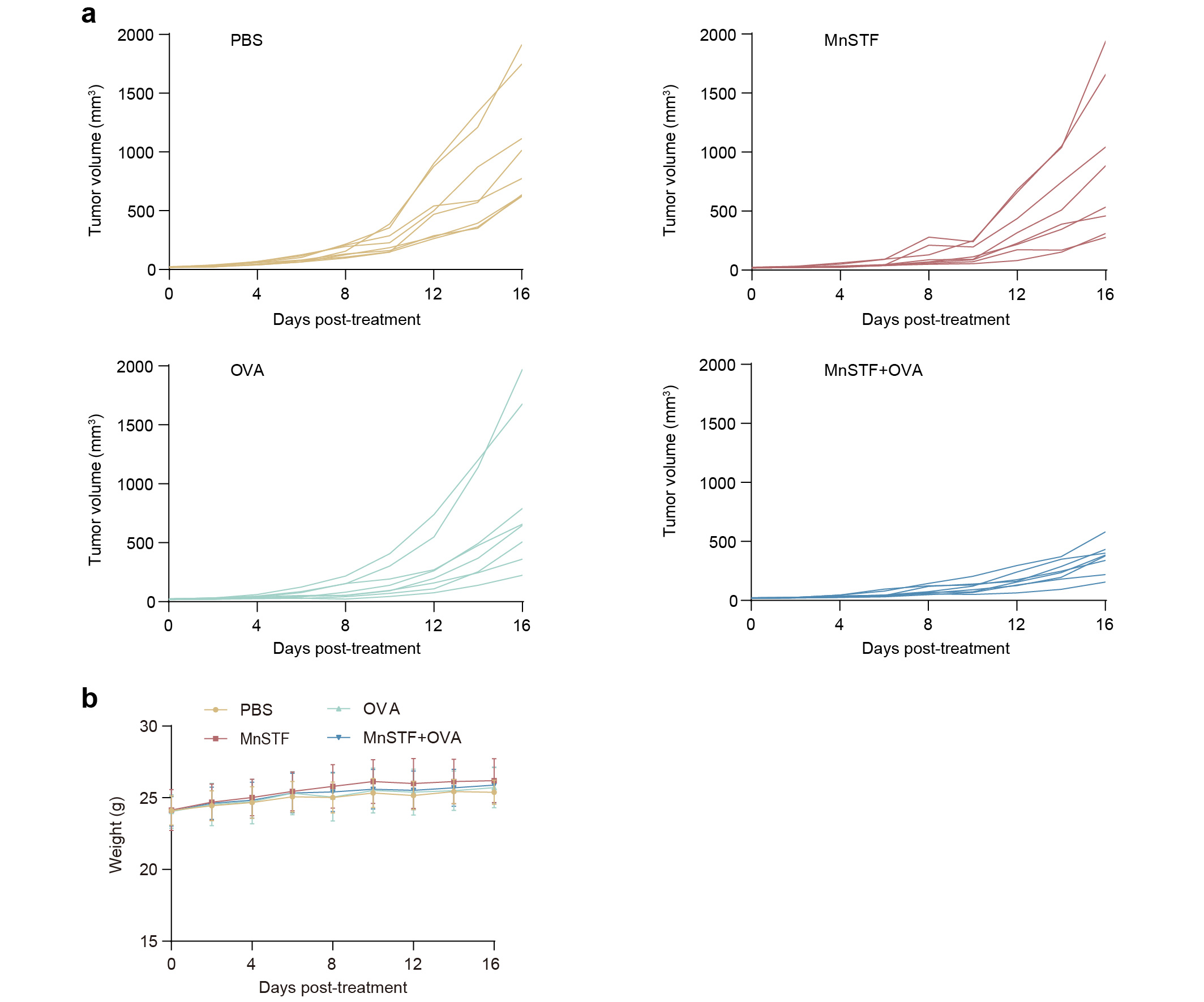
**Supplementary Fig. 12 |**  (**a**) Growth curves of individual B16F10-OVA tumors in the PBS, MnSTF, OVA or MnSTF + OVA groups (n = 8). (**b**) Body weight changes of mice in the PBS, MnSTF, OVA, or MnSTF + OVA groups (n = 8).


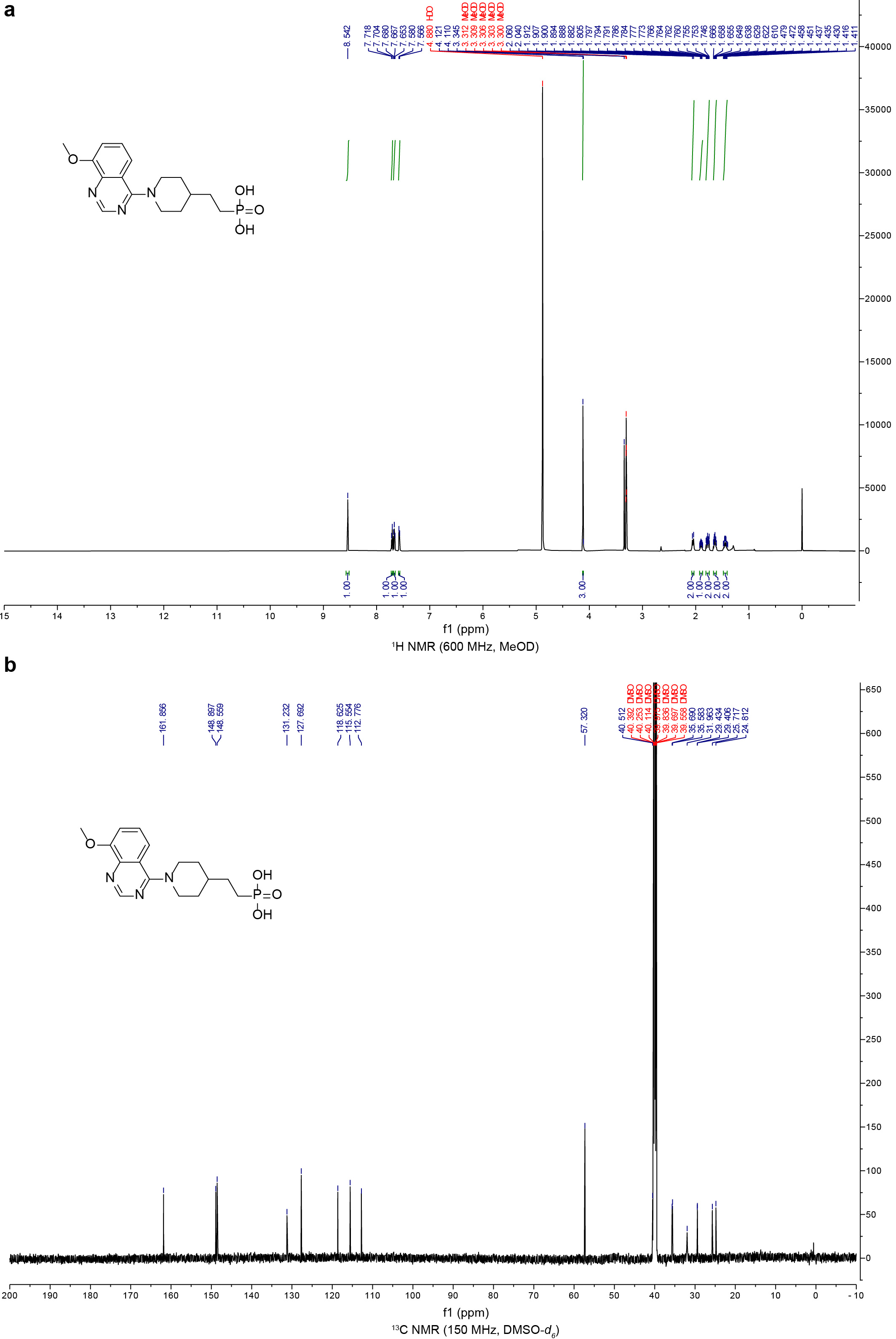


**Supplementary Fig. 13 |** ^1^NMR and ^13^C NMR spectra of P-[2-[1-(8-Methoxy-4-quinazolinyl)-4-piperidinyl]ethyl]phosphonic acid (**STF-1623**).


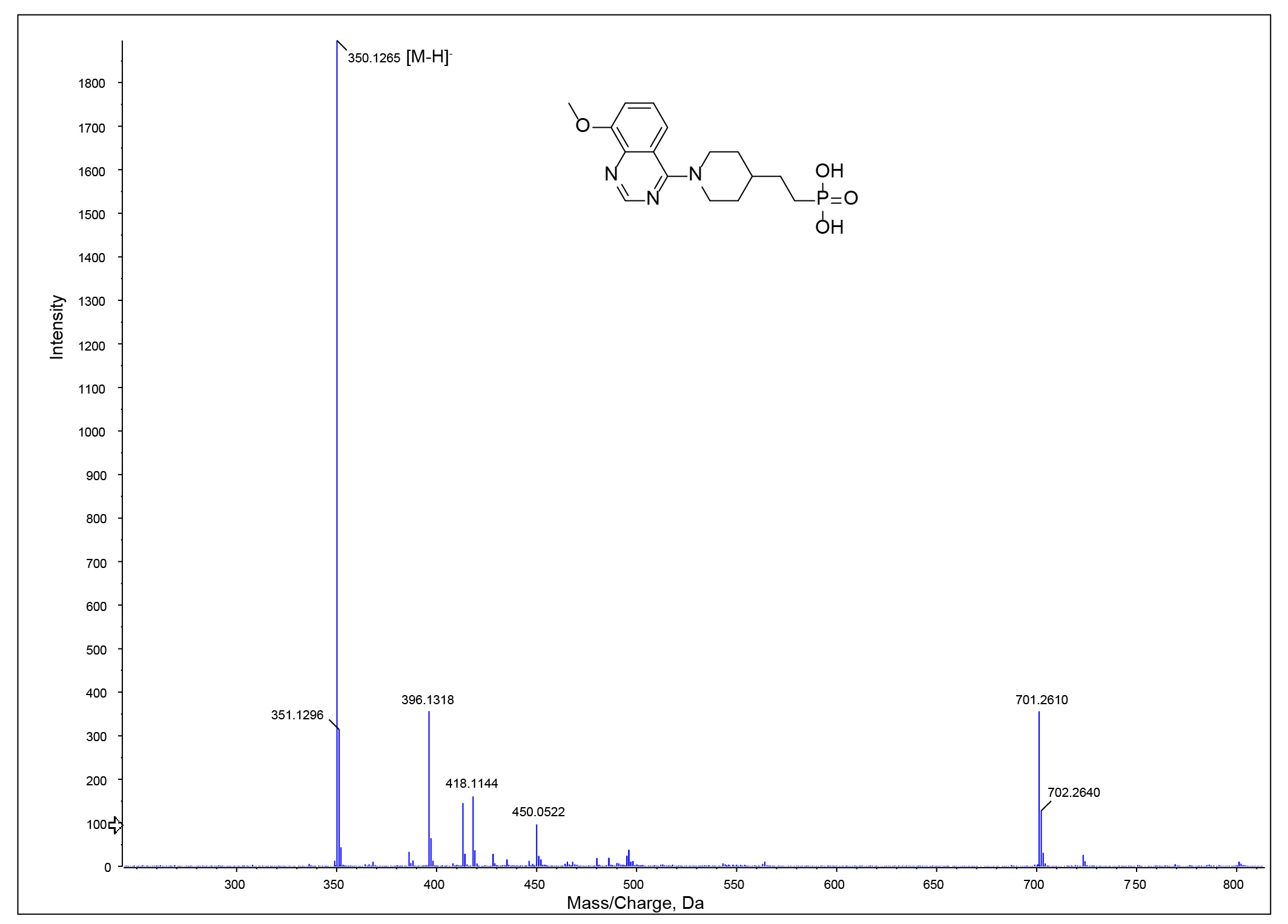


**Supplementary Fig. 14 |** HRMS spectrum of P-[2-[1-(8-Methoxy-4-quinazolinyl)-4-piperidinyl]ethyl]phosphonic acid (**STF-1623**).


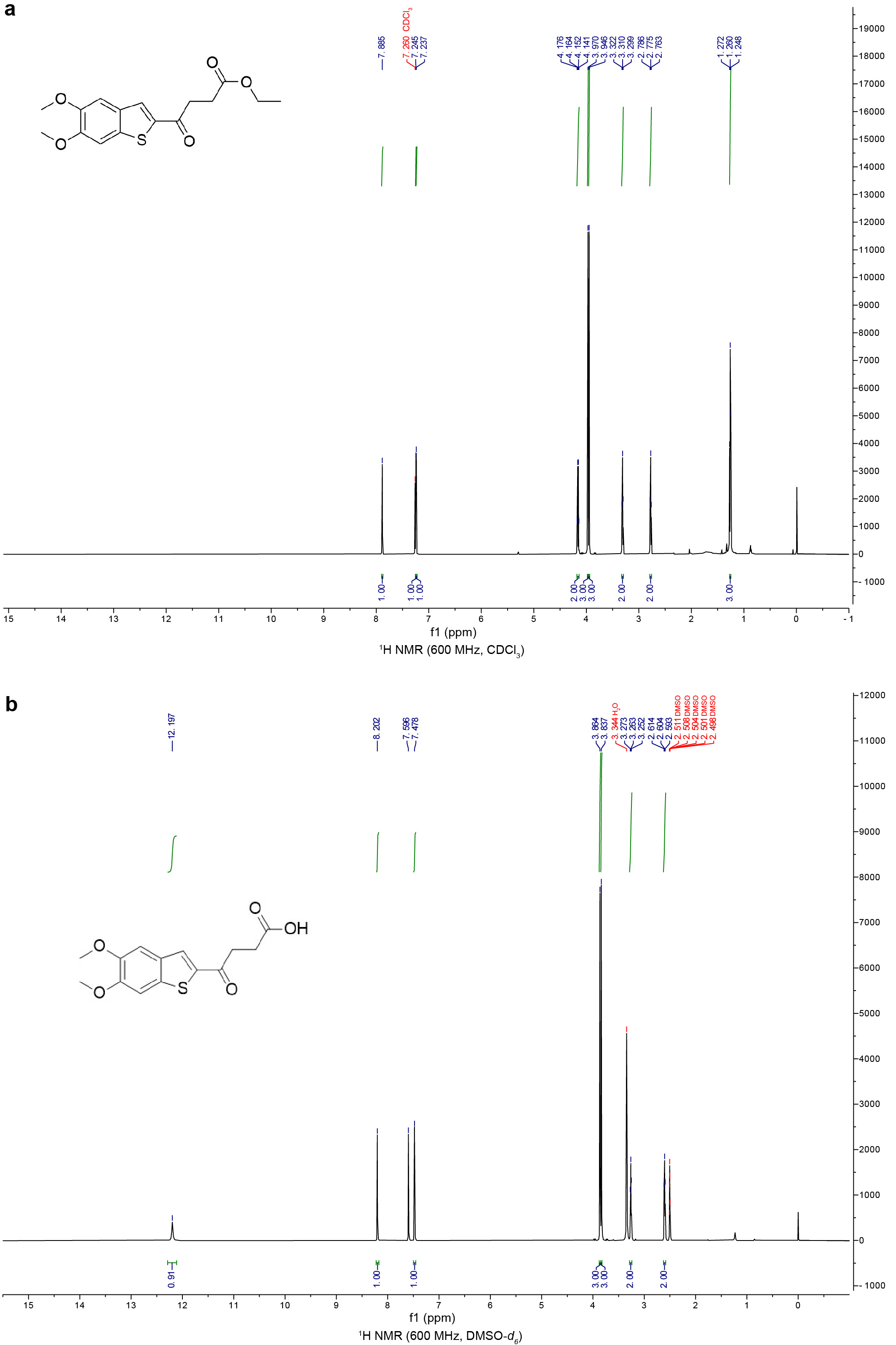


**Supplementary Fig. 15 |** ^1^NMR spectra of ethyl 4-(5,6 dimethoxybenzo[b]thiophen-2-yl)-4-oxobutanoate (**a**) and 4-(5,6-dimethoxybenzo[b]thiophen 2-yl)-4-oxobutanoic acid (**MSA-2**) (**b**).


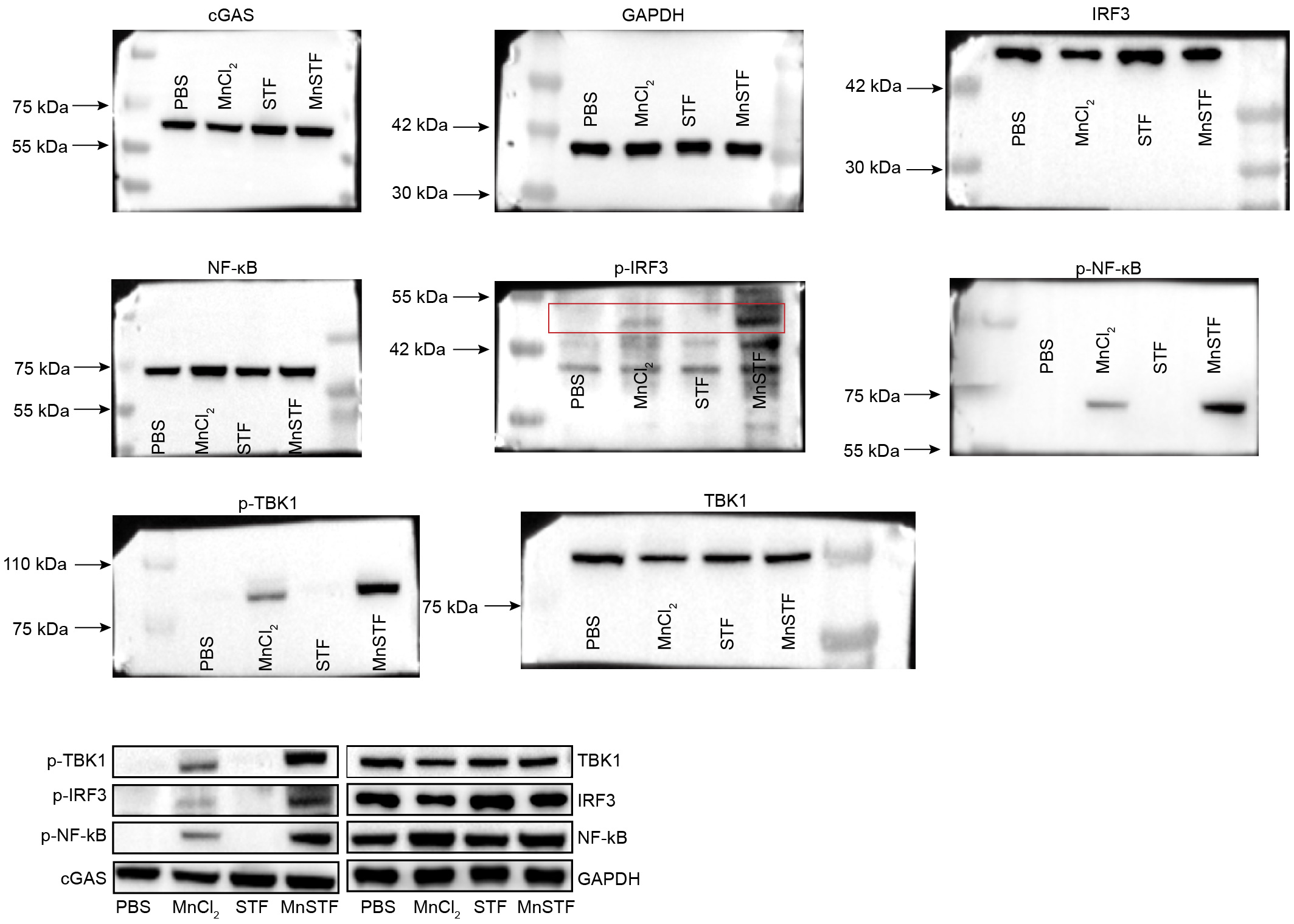


**Supplementary Fig. 16 |** The raw data from the immunoblot experiments as seen in **Fig. 3b**.

**References**

[1] A. R. Norman, M. N. Yousif, C. S. P. McErlean, *Org. Chem. Front.* **2018**, *5*, 3267.

[2] Y. L. Sun, M. M. Chen, Y. Y. Han, W. Q. Li, X. Y. Ma, Z. H. Shi, Y. Zhou, L. Xu, L. Yu, Y. X. Wang, J. H. Yu, X. X. Diao, L. H. Meng, S. L. Xu, *J. Med. Chem.* **2024**, *67*, 3986.

[3] J. A. Carozza, V. Böhnert, K. C. Nguyen, G. Skariah, K. E. Shaw, J. A. Brown, M. Rafat, R. von Eyben, E. E. Graves, J. S. Glenn, M. Smith, L. Li, *Nat. Cancer* **2020**, *1*, 184.

[4] B. S. Pan, S. A. Perera, J. A. Piesvaux, J. P. Presland, G. K. Schroeder, J. N. Cumming, B. W. Trotter, M. D. Altman, A. V. Buevich, B. Cash, S. Cemerski, W. Chang, Y. P. Chen, P. J. Dandliker, G. Feng, A. Haidle, T. Henderson, J. Jewell, I. Kariv, I. Knemeyer, J. Kopinja, B. M. Lacey, J. Laskey, C. A. Lesburg, R. Liang, B. J. Long, M. Lu, Y. H. Ma, E. C. Minnihan, G. O'Donnell, R. Otte, L. Price, L. Rakhilina, B. Sauvagnat, S. Sharma, S. Tyagarajan, H. Woo, D. F. Wyss, S. Xu, D. J. Bennett, G. H. Addona, *Science* **2020**, *369*, 935.
